# Data Interoperability in COVID-19 Vaccine Trials: Methodological Approach in the VACCELERATE Project

**DOI:** 10.2196/65590

**Published:** 2025-03-07

**Authors:** Salma Malik, Zoi Pana Dorothea, Christos D Argyropoulos, Sophia Themistocleous, Alan J Macken, Olena Valdenmaiier, Frank Scheckenbach, Elena Bardach, Andrea Pfeiffer, Katherine Loens, Jordi Cano Ochando, Oliver A Cornely, Jacques Demotes-Mainard, Sergio Contrino, Gerd Felder

**Affiliations:** 1 European Clinical Research Infrastructure Network Paris France; 2 School of Medicine European University Cyprus Nicosia Cyprus; 3 Chemical Engineering Department King Fahd University of Petroleum and Minerals Dhahran Saudi Arabia; 4 Centre for Experimental Pathogen Host Research University College Dublin School of Medicine National University of Ireland Dublin Ireland; 5 Centre of Excellence for Health, Immunity and Infections, Rigshospitalet University of Copenhagen Copenhagen Denmark; 6 Clinical Trials Centre Cologne Faculty of Medicine University of Cologne Cologne Germany; 7 Laboratory of Medical Microbiology VAXINFECTIO, Faculty of Medicine and Health Sciences University of Antwerp Antwerp Belgium; 8 Microbiology Section, Department of Pharmaceutical Sciences and of Health Faculty of Pharmacy Universidad San Pablo-Centro de Estudios Universitarios Madrid Spain; 9 CECAD, CIO ABCD, ECMM, CMMC Faculty of Medicine and University Hospital Cologne University of Cologne Cologne Germany

**Keywords:** interoperability, metadata, data management, clinical trials, protocol, harmonization, adult, pediatric, systems, standards

## Abstract

**Background:**

Data standards are not only key to making data processing efficient but also fundamental to ensuring data interoperability. When clinical trial data are structured according to international standards, they become significantly easier to analyze, reducing the efforts required for data cleaning, preprocessing, and secondary use. A common language and a shared set of expectations facilitate interoperability between systems and devices.

**Objective:**

The main objectives of this study were to identify commonalities and differences in clinical trial metadata, protocols, and data collection systems/items within the VACCELERATE project.

**Methods:**

To assess the degree of interoperability achieved in the project and suggest methodological improvements, interoperable points were identified based on the core outcome areas—immunogenicity, safety, and efficacy (clinical/physiological). These points were emphasized in the development of the master protocol template and were manually compared in the following ways: (1) summaries, objectives, and end points in the protocols of 3 VACCELERATE clinical trials (EU-COVAT-1_AGED, EU-COVAT-2_BOOSTAVAC, and EU-COVPT-1_CoVacc) against the master protocol template; (2) metadata of all 3 clinical trials; and (3) evaluations from a questionnaire survey regarding differences in data management systems and structures that enabled data exchange within the VACCELERATE network.

**Results:**

The noncommonalities identified in the protocols and metadata were attributed to differences in populations, variations in protocol design, and vaccination patterns. The detailed metadata released for all 3 vaccine trials were clearly structured using internal standards, terminology, and the general approach of Clinical Data Acquisition Standards Harmonisation (CDASH) for data collection (eg, on electronic case report forms). VACCELERATE benefited significantly from the selection of the Clinical Trials Centre Cologne as the sole data management provider. With system database development coordinated by a single individual and no need for coordination among different trial units, a high degree of uniformity was achieved automatically. The harmonized transfer of data to all sites, using well-established methods, enabled quick exchanges and provided a relatively secure means of data transfer.

**Conclusions:**

This study demonstrated that using master protocols can significantly enhance trial operational efficiency and data interoperability, provided that similar infrastructure and data management procedures are adopted across multiple trials. To further improve data interoperability and facilitate interpretation and analysis, shared data should be structured, described, formatted, and stored using widely recognized data and metadata standards.

**Trial Registration:**

EudraCT 2021-004526-29; https://www.clinicaltrialsregister.eu/ctr-search/trial/2021-004526-29/DE/; 2021-004889-35; https://www.clinicaltrialsregister.eu/ctr-search/search?query=eudract_number:2021-004889-35; and 2021-004526-29; https://www.clinicaltrialsregister.eu/ctr-search/search?query=eudract_number:2021-004526-29

## Introduction

Interoperability is defined as the ability of different information systems, devices, or applications to connect in a coordinated manner within and across organizational boundaries, enabling stakeholders to access, exchange, and cooperatively use data with the goal of optimizing the health of individuals and populations [1].

Interoperability standards provide a common language and set of expectations that enable interoperability between systems and devices. These standards allow clinicians, laboratories, hospitals, pharmacies, and patients to seamlessly share data, regardless of the application or market supplier, thereby improving the coordination and delivery of health care [1].

The health interoperability ecosystem comprises individuals, systems, and processes that aim to share, exchange, and access all forms of health information, including discrete, narrative, and multimedia data. Potential stakeholders in this ecosystem include individuals, patients, providers, hospitals/health systems, researchers, payers, suppliers, and systems, all of whom contribute to the creation, exchange, and use of health information and data. When data are structured according to international standards, they become much easier to analyze, reducing the efforts required for data cleaning and preprocessing. Data standards are not only essential for efficient data processing but are also fundamental to ensuring the interoperability of the data itself [2]. The greater use of data standards is crucial to the success of data sharing. Without such standards, shared data are difficult to interpret with certainty, significantly more time-consuming to process, and therefore more expensive to collect. Standards can be applied to data item definitions and codes, controlled vocabularies for categories, and the structuring and exchange of data. File formats used for storing and transferring data should also be standardized to simplify data processing [3]. Clinical trial datasets should always include metadata describing the characteristics of each data item (eg, type, code, name, and possibly an ontology reference), along with details of the trial’s schedule and design. Datasets should be made available for sharing in 1 or more standardized file formats that are compatible with a wide variety of systems [3].

In January 2021, the European Commission launched VACCELERATE—the European Corona Vaccine Trial Accelerator Platform [4] (GA 101037867)—to coordinate phases II and III of COVID-19 vaccine trials across Europe. VACCELERATE aimed to serve as a single-entry point for multicountry vaccine trials in Europe and to address the need for greater inclusion of underrepresented populations in vaccine trials. One of the fundamental objectives of VACCELERATE was to promote the sharing, interoperability, and FAIRness (Findable, Accessible, Interoperable, and Reusable) of data generated through the VACCELERATE network. This included ensuring that data sharing and future reuse were considered by investigators, the scientific community, and study sponsors. The objective of this study was to assess the degree of commonalities and differences among clinical trial metadata, protocols, and data collection systems/items in VACCELERATE consortium projects [5-8]. Additionally, the study aimed to explore the reasons why interoperability was sometimes limited or failed to occur. It also sought to identify areas where interoperability was relatively straightforward and to propose structures and systems that could enhance interoperability in the future, including for data generated outside the trials funded under the VACCELERATE platform [9-11].

EU-COVAT-1_AGED (EudraCT: 2021-004526-29) is a multinational, phase II, randomized, adaptive protocol designed to evaluate the immunogenicity and reactogenicity of different COVID-19 vaccines in adults aged ≥75 years who have already been vaccinated against SARS-CoV-2. It is the first of 3 VACCELERATE phase II COVID-19 vaccine trials and focuses on the immunogenicity and reactogenicity of vaccines in older adults (≥75 years), addressing a critical demographic that is often underrepresented in vaccine studies [9].

EU-COVAT-2_BOOSTAVAC (EudraCT: 2021-004889-35) is an international, multicenter, phase II, randomized, adaptive protocol designed to evaluate the need for, optimal timing of, and immunogenicity of administering a booster messenger RNA (mRNA) vaccination dose against SARS-CoV-2 in the general population (aged 18+ years) already vaccinated against SARS-CoV-2. This trial investigates booster vaccination strategies in the general adult population, providing insights into optimal timing and immunological outcomes for sustaining immunity [9].

EU-COVPT-1_COVACC (EudraCT: 2021-004526-29) is a phase II, comparative, randomized trial designed to evaluate the impact of a reduced COVID-19 mRNA vaccination regimen on immunological responses and reactogenicity in pediatric patients with prior SARS-CoV-2 immunity. This trial investigates reduced-dose regimens in pediatric populations, contributing to the understanding of tailored vaccination approaches for children [9].

Together, these trials represent a comprehensive investigation across key demographic groups—older adults, the general population, and children—using adaptive and multinational protocols that address the challenges of rapid vaccine development and deployment during pandemics.

## Methods

### Overview

The degree of commonality and interoperability among data was assessed across 3 levels: clinical trial protocols, metadata, and data systems and management.

### Interoperability Among the VACCELERATE Clinical Trial Protocols and Master Protocol Template

**Figure 1:**
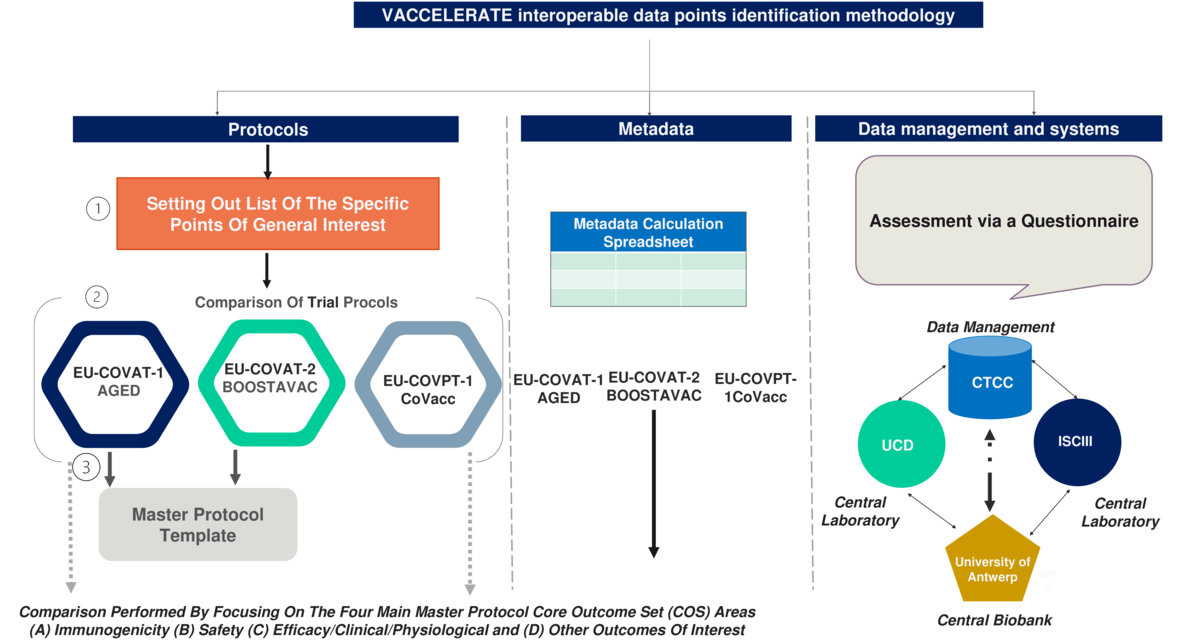
Methodology for the identification of the interoperable data points. CTCC: Clinical Trials Centre Cologne; UCD: University College Dublin.

The process began with generating a list of the main points and parameters of general interest (eg, type of trial, randomization, primary and secondary aims, and safety) across the 3 specific clinical trial protocols: EU-COVAT-1_AGED, EU-COVAT-2_BOOSTAVAC, and EU-COVPT-1_COVACC [9,10]. The second step involved comparing the 3 trial protocols to identify interoperable data points based on this list of key parameters. In step 3, the outcomes derived from the adult trial protocols were compared against the VACCELERATE adult master protocol (Figure 1). This comparison focused on 4 core areas of the master protocol template’s core outcome set (COS): (1) immunogenicity, (2) safety, (3) efficacy/clinical/physiological, and (4) other outcomes of interest. Differences and similarities among the protocols were identified manually.

### Metadata Interoperability of the VACCELERATE Clinical Trials

All data management (DM) processes, including the design of study databases for all 3 VACCELERATE clinical trials, were carried out by the Clinical Trials Centre Cologne (CTCC). The DM team at the CTCC developed the Study Database Specification (DB Spec) using an Excel (Microsoft Corporation) workbook. This workbook included metadata for defining the study database, case report forms (CRFs), edit checks, and a data dictionary. For each CRF study, the workbook contained an individual worksheet specifying database details such as field names, field response types, and formats. Additionally, the detailed metadata for all 3 vaccine trials were systematically developed using internal standards, terminology, and the general approach of Clinical Data Acquisition Standards Harmonization (CDASH) [12] for data collection, such as on electronic CRFs (eCRFs; Table 1). For instance, questions were categorized into relevant Clinical Data Interchange Standards Consortium (CDISC) [13] domains, along with prefixes for adverse events (AEs), concomitant medications, vital signs, protocol deviation, etc, while standard CDISC suffixes were applied to indicate question types.

**Table 1:** Example of CDASH^a^ data items within EU-COVAT-1_AGED.

Caption	Variable	Length/format	Type	Explanation/code list
Sample collected?	LBPERF	N/A^b^	Category	YES=yes, NO=no
Reason for sample not collected	LBREASND	200	Character	N/A
Date of collection	LBDTC	N/A	Date	N/A
Name of Lab	LBNAME	N/A	Combo box	N/A
Test name	LBTEST	30	Label	Laboratory test names: blood urea nitrogen, creatinine, aspartate transaminase, alanine transaminase, total bilirubin, and alkaline phosphatase
Result	LBORRES	99999.99	Float	Laboratory test result
Unit	LBORRESU	N/A	Combo box	Laboratory test unit
Reference range lower limit	LBORNRLO	10	Character	Laboratory test min
Reference range upper limit	LBORNRHI	10	Character	Laboratory test max
Export unit	LBEXUNIT	10	Character	Laboratory test export unit
Clinically significant	LBCLSIG	N/A	Category	YES=yes, NO=no

^a^CDASH: Clinical Data Acquisition Standards Harmonization.

^b^N/A: not applicable.

Although the databases were generated by a single center and coordinated by the same individual, it was valuable to evaluate the degree of standardization, commonality, and data interoperability among the DB Spec of all 3 trials. To achieve this, a manual comparison of the Excel DB Spec from all 3 VACCELERATE clinical trials was conducted (Figure 1). This comparison focused on the main core areas—immunogenicity, safety, efficacy/clinical/physiological—emphasizing the development of the master protocol template.

The other variables were not considered due to differences in demographic details and treatment approaches among the 3 groups (older adults, adult, and pediatric). Metadata items such as data format, data dictionary name, response type, item text, required field indicator, and review groups were compared for consistency and discrepancies. Once identified, the next step was to analyze the rationale behind the observed consistencies and discrepancies among the metadata items.

### Interoperability in Data Management and Systems

Interoperability in DM and systems was assessed through a questionnaire (Multimedia Appendix 1) distributed to the central laboratories: the University College Dublin (UCD) Centre for Experimental Pathogen Host Research (CEPHR, Dublin), Instituto de Salud Carlos III (ISCIII, Madrid), and the central biobank (UZA/UAntwerpen Biobank, Antwerp University Hospital). The questionnaire aimed to gather their experiences working with the VACCELERATE DM systems (Figure 1). UZA/UAntwerpen provided an overview of samples received from local sites (shipment manifest) and transferred to the research laboratories (transport manifest) and the clinical trial center. CEPHR and ISCIII, as research laboratories, performed the specified analyses on samples received from the central biobank. After completing the analyses, the research laboratories sent the results to the CTCC.

The questionnaire was designed to maximize accessibility and ease of use for site staff by categorizing the questions into broad areas while allowing flexibility through free-text response options: (1) laboratory procedures and documentation, (2) sample handling and storage data, (3) data transfer, and (4) general laboratory DM. Based on the information gathered through the questionnaire, a descriptive analysis was conducted to examine how each site handled, stored, and transferred data, as well as how their processes evolved within the VACCELERATE network. Furthermore, the analysis evaluated potential factors and new concepts required to harmonize and standardize the VACCELERATE DM systems.

The DM documents—external DM plans, edit check plans, CRF completion requirements, and DB Specs—developed for the clinical trials by the CTCC were reviewed. Guided by the questionnaire, a semistructured interview was conducted with the CTCC data manager.

### Ethical Considerations

The 3 VACCELERATE COVID-19 vaccine trials (EU-COVAT-1_AGED, EU-COVAT-2_BOOSTAVAC, and EU-COVPT-1_COVACC) received favorable ethics opinions and approval from the national competent authorities in the participating countries (State Medicines Control Agency, Norwegian Medicines Agency, Spanish Agency of Medicines and Health Products, Health Products Regulatory Authority Ireland, and Paul-Ehrlich-Institut for the EU-COVAT-1 AGED trial; Health Products Regulatory Authority Ireland, Norwegian Medicines Agency, Swedish Medical Products Agency, The Spanish Agency of Medicines and Medical Devices, Federal Agency for Medicines and Health Products, and Paul-Ehrlich-Institut for the EU-COVAT-2 BOOSTAVAC trial; and Netherlands – Competent Authority, Norwegian Medicines Agency, Swedish Medical Products Agency, Hellenic National Organization for Medicines, and Paul-Ehrlich-Institut for the EU-COVPT-1 COVACC trial). Written informed consent was obtained from all participants before any trial procedures. These trials were conducted in accordance with the International Conference on Harmonization for Good Clinical Practice (ICH-GCP) guidelines and complied with the Declaration of Helsinki at all times. Furthermore, the provisions of the EU General Data Protection Regulation (GDPR) 2016/679 were strictly observed to ensure data protection. All investigational materials and data were pseudonymized in compliance with data protection regulations before undergoing scientific processing.

## Results

### Interoperability Among the Three Clinical Trial Protocols and Master Protocol

Our harmonization and standardization efforts began with a comparison of the 3 clinical trial protocols against the COS of the master protocol. The COS includes all appropriate measurements necessary to ensure standardization, efficiency, and uniformity in the conduct of clinical trials. Four main COS areas were adopted for phase II and III studies: (1) immunogenicity, (2) safety, (3) efficacy/clinical/physiological, and (4) other outcomes of interest.

**Figure 2:**
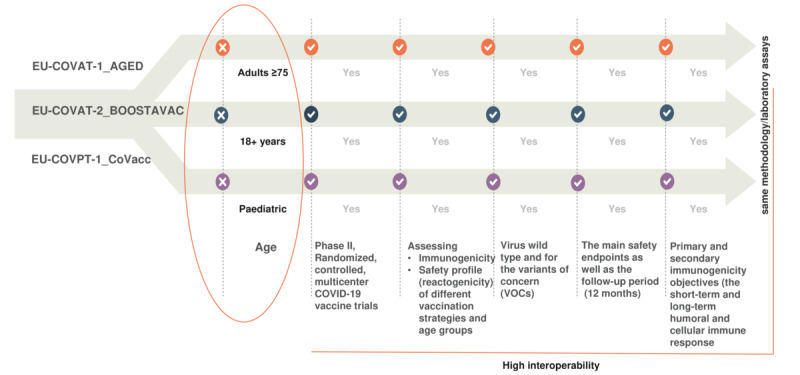
Interoperability between the 3 specific clinical trial protocols (summary).

The harmonization process resulted in the creation of Tables 2-5, which illustrate the interoperability between the master protocol and the 3 clinical trial protocols based on the COS areas. A summary of the outcomes is presented in Figure 2 (see Table S1 in Multimedia Appendix 2). Humoral and cellular responses to COVID-19 vaccines were assessed in a similar manner across all trials, considering both vaccine and host characteristics (Table 2), although minor adjustments to time points were incorporated into the pediatric protocol.

**Table 2:** Core area: immunogenicity (core outcome set).

Objective	Outcome	Method of measurement
To evaluate humoral response to vaccination against wild-type SARS-CoV-2 and variants of interest—(1) short term: 7, 14, and 28 days after randomization and (2) long term: 3, 6, 9, and 12 months after randomization	Binding antibodies titers (IgAa, IgG, and IgM) against SARS-CoV-2 nucleocapsid, receptor-binding domain, spike 1, and spike 2 viral proteins. SARS-CoV-2 neutralization	Immunoassay (enzyme-linked immunosorbent assay, electrochemiluminescence immunoassay, or chemiluminescence immunoassay); expressed as BAU/ml (geometric mean titers). Neutralizing antibody titers to live wild-type SARS-CoV-2 plaque reduction neutralization test (PRNT50) assay, and pseudovirion-expressing S protein from different variants as measured by NT50 or flow cytometry–based microneutralization assay; expressed as IU/ml
To evaluate the cellular immune response to vaccination against wild-type SARS-CoV-2 and variants of interest—(1) short term: 7, 14, and 28 days after randomization); and (2) long term: 3, 6, 9, and 12 months after randomization)	Th1b and Th2 immune responses	Flow cytometry after SARS-CoV-2 S protein peptide stimulation of peripheral blood mononuclear cells and intracellular staining, including CD4+c/CD8+ interferon-gamma, interleukin-2, tumor necrosis factor-alpha, interleukin-4, interleukin-5, interleukin-13, and other Th1/Th2 markers.

^a^Ig: immunoglobulin.

^b^Th: helper T cell.

^c^CD: cluster of differentiation.

The early detection of serious AEs (SAEs) can be vital for the protection of the participants and via the interoperability of safety data, an overall better general view of the patient’s clinical data could be achieved, resulting in the reduction of any potential risk of harms or adverse impacts and higher clinical safety. The developing safety profile in the trials is in a sufficiently similar motive and includes all the required information about reporting solicited, unsolicited, serious, and medically attended AEs, as shown in Table 3. By contrast, the definitions of the COVID-19 disease (eg, asymptomatic infection, confirmed and reinfection cases, the severity of signs and symptoms) are not reported in the protocols, even though it is referred to as part of the trial dataset outcomes (Table 4). Finally, the profile of immunophenotyping of immune responses (at the cellular level) and genotypic testing of breakthrough infections (vaccine immunogenicity) were also determined in the protocols, enhancing interoperability among them (Table 5). It should be noted that we did not present herein the comparison of pediatric protocol versus pediatric master protocol because the former is currently under review process.

**Table 3:** Core clinical area: safety data (core outcome set).

Outcome	Method of measurement
Solicited local and systemic AEs^a^ for 7 days after each vaccination.	AEs are collected in a diary and will be entered into the electronic case report form (see all potential adverse reactions in Multimedia Appendix 2).
Unsolicited AEs after each vaccination	AEs are collected during visits upon an open question by the investigator up to the end of the trial.
SAEs^b^	SAEs collected throughout the study (from first vaccination until the end of the study)
Medically attended adverse events	Medically attended adverse events from the day of vaccination until the end of the study.

^a^AE: adverse event.

^b^SAE: serious adverse event.

**Figure 3:**
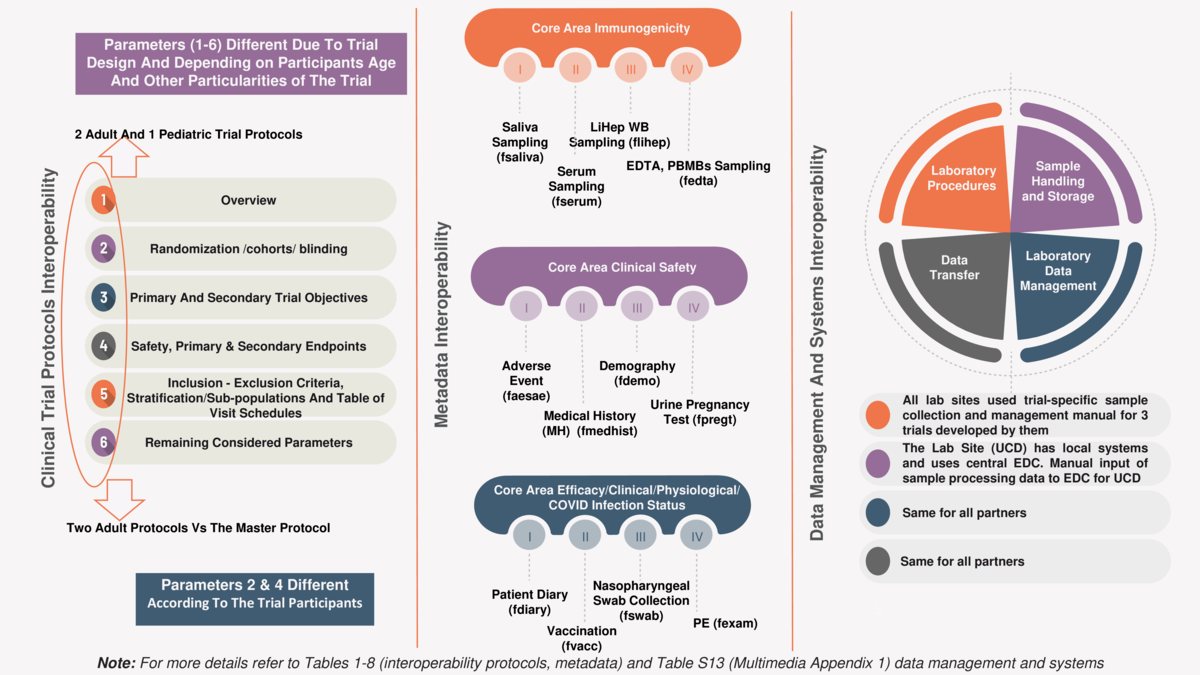
Noncommonality parameters identified in the VACCELERATE projects (protocols, metadata and data management, and systems). EDC: Electronic Data Capture; EDTA: ethylenediaminetetraacetic acid; LiHep: lithium heparin; PBMC: peripheral blood mononuclear cell; PE: physical examination; UCD: University College Dublin; WB: whole blood.

**Figure 4:**
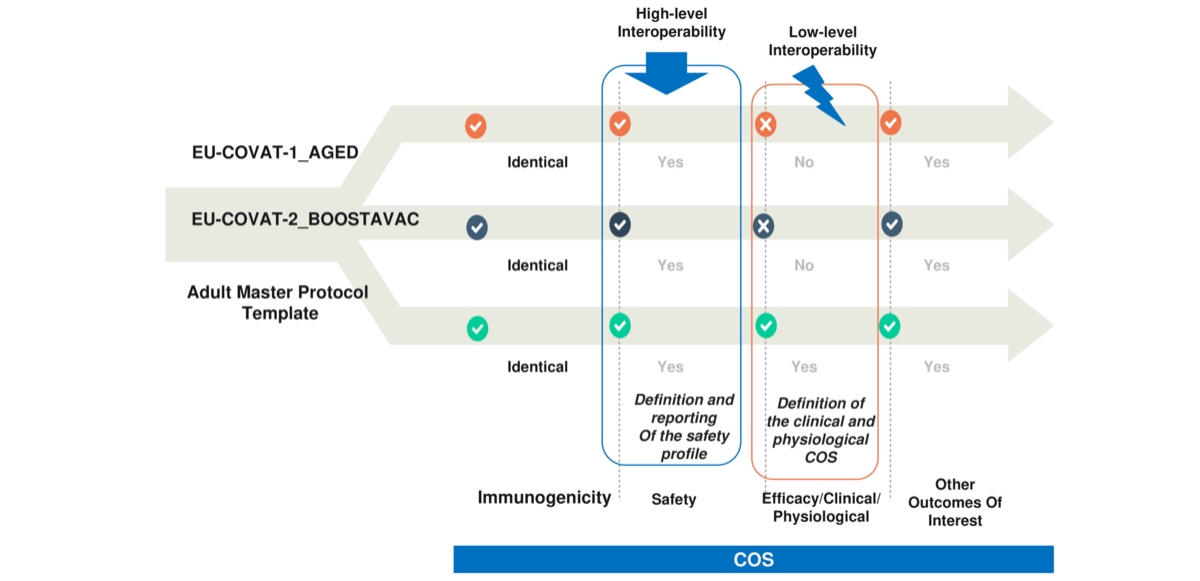
COS interoperability between the vaccine trial and master protocols.

Several parameters were considered to enable and enhance interoperability across the developed COVID-19 vaccine trials and master protocols (Figures 3 and 4). It is well-known that the lack of standardization in data processing exacerbates inhomogeneity in raw data. This often results in errors and significant time delays, reducing the reusability and interoperability of data within protocols. To address this, the various protocol formats were homogenized by manually identifying discrepancies and commonalities among selected parameters (see Tables S2-S13 in Multimedia Appendix 2). The selection of parameters was based on their relevance to the trials. Parameters present in only 1 protocol were excluded, while efforts were made to promote the consistent use of the same parameters across adult trials.

Concealment (blinding) of treatment group allocation is not anticipated for the current trials, while both adult trials support vaccine booster strategies. All clinical trials assess reactogenicity symptoms and the immunogenicity (efficacy) profile of COVID-19 vaccines (following a fourth dose) to inform potential optimized vaccine strategies. The secondary objectives include evaluating immune response for up to 12 months (see Tables S2-S4 in Multimedia Appendix 2).

The safety aims and the increase in antibody titer were found to be similar across all trials, as were the primary end points in the adult population protocols, due to the nature of the BOOSTAVAC trial (see Table S5 in Multimedia Appendix 2). By contrast, stratification/subpopulation, inclusion, and exclusion criteria among volunteers are directly influenced by the participants’ age and other trial-specific factors (eg, vaccine scheme, gender, immune status; see Table S6 in Multimedia Appendix 2). Additionally, patients’ insurance and consent forms are addressed in all protocols, and the sample size calculation was reported based on the specifics of each protocol (see Table S7 in Multimedia Appendix 2).

**Table 4:** Core clinical area: efficacy/clinical/physiological (core outcome set).

Set of data and definition			Explanation
**Confirmed COVID-19 disease**			
	COVID-19 disease	Molecularly confirmed COVID-19 is defined as a positive SARS-CoV-2 viral RNA result using a PCR^a^-based or other molecular diagnostic test or positive antigen test. (See Multimedia Appendix 2 for the definition of confirmed COVID-19 disease.)	
**Asymptomatic SARS-CoV-2 infection**			
	Asymptomatic SARS-CoV-2 infection^b^	The same as above but without symptoms typical for/suspicious of COVID-19	
**Severity of COVID-19 disease**			
	Asymptomatic SARS-CoV-2 infection or COVID-19 disease (presence and severity of signs and symptoms of COVID-19 disease)	Signs and symptoms as measured by symptoms of infection with COVID-19^c^ at first evaluation and at maximum severity: mild, moderate, and severe COVID-19	
**Severe COVID-19 disease**			
	COVID-19 disease fulfilling criteria for intensive care unit admittance	Clinical records	
**All-cause mortality**			
	All-cause mortality: (1) at day 30 from randomization and (2) time from randomization	Clinical records	
**Disease (or infection) duration**			
	Time to 2019-nCoV RT-PCR^d^ negativity (days)	Negativity: 2 consecutive negative results (sampling interval of at least 24 hours) of the 2019-nCoV nucleic acids tests of respiratory pathogens	
	Length (days) of hospital stay	Discharge standards: (1) normal body temperature for more than 3 days; (2) significant recovery from respiratory symptoms; (3) lung imaging showing obvious absorption and recovery of the acute exudative lesion; and (4) negativity of nucleic acids tests performed twice.	
	Length of intensive care unit stay	N/A^e^	
**Reinfection**			
	Confirmed COVID-19 reinfection	Molecularly confirmed COVID-19 is defined as a positive SARS-CoV-2 viral RNA result using a PCR-based or another molecular diagnostic test or positive antigen test. (See Multimedia Appendix 2 for the definition of confirmed COVID-19 disease.)	
**Time to reinfection**			
	Long COVID syndrome	WHO definition^b^ [14,15]	
	Multisystem inflammatory syndrome in children	MIS-C^f^ WHO^g^ definition	

^a^PCR: polymerase chain reaction.

^b^See [15].

^c^Per case definition for moderate to severe/critical COVID-19.

^d^RT-PCR: reverse transcription polymerase chain reaction.

^e^N/A: not applicable.

^f^MIS-C: multisystem inflammatory syndrome in children associated with COVID-19.

^g^WHO: World Health Organization.

**Table 5:** Other outcomes of interest.^a^

Set of data	Outcome and methods
Immunophenotyping of immune responses to vaccination	Detailed immunophenotyping of immune responses, including metabolomics, proteomics, and transcriptomics
Genotyping of breakthrough infections	Whole genome sequencing of isolated SARS-CoV-2 viral strains detected in breakthrough infections

^a^Per the CoVacc trial protocol, Participants will be informed that leftover blood samples, remaining after all study-related testing is completed, may be stored indefinitely for potential future research purposes (eg, exploratory immunology). This may include genotypic testing of genetic polymorphisms relevant to vaccine immunogenicity. Participants will have the option to decide whether they permit the future use of any leftover samples. With the participants’ informed consent, any remaining cells and plasma will be frozen indefinitely for future analyses related to COVID-19 and other coronavirus-related diseases or vaccine-related responses. If a participant chooses not to allow this, all leftover samples belonging to that participant will be discarded at the end of the trial.

We also conducted a comparison of the 2 adult trials with the master protocol (see Tables S8-S13 in Multimedia Appendix 2). The primary aim of the adult trials is to evaluate the immunogenicity and reactogenicity (safety) of vaccine booster strategies and homologous mRNA vaccination for COVID-19 at different time intervals (see Tables S8 and S9 in Multimedia Appendix 2).

Both the adult and master protocols include the relevant subprotocols, cohorts, and descriptions of the randomization process, while blinding is not anticipated in the adult trials. All protocols support phase II trials and share similar primary objectives. The primary and secondary objectives/end points (including exploratory ones) constitute a nearly identical core dataset. The secondary trial objectives for immunogenicity and reactogenicity focus on the immune response and associated signs and symptoms over up to 12 months (see Tables S9 and S10 in Multimedia Appendix 2).

The reporting periods for unsolicited and solicited AEs, as well as the rates of SAEs, are identical across the adult and master protocols. However, discrepancies in inclusion and exclusion criteria are directly influenced by the trial participants (see Tables S11 and S12 in Multimedia Appendix 2). The treatment duration and follow-up period for the studies are identical in both the adult trials and the master protocol, extending up to 12 months. All clinical trials are conducted in accordance with the ICH-GCP, including the use of appropriate consent forms and patient insurance, based on the allocated treatment group (see Table S13 in Multimedia Appendix 2).

### Metadata Interoperability

**Figure 5:**
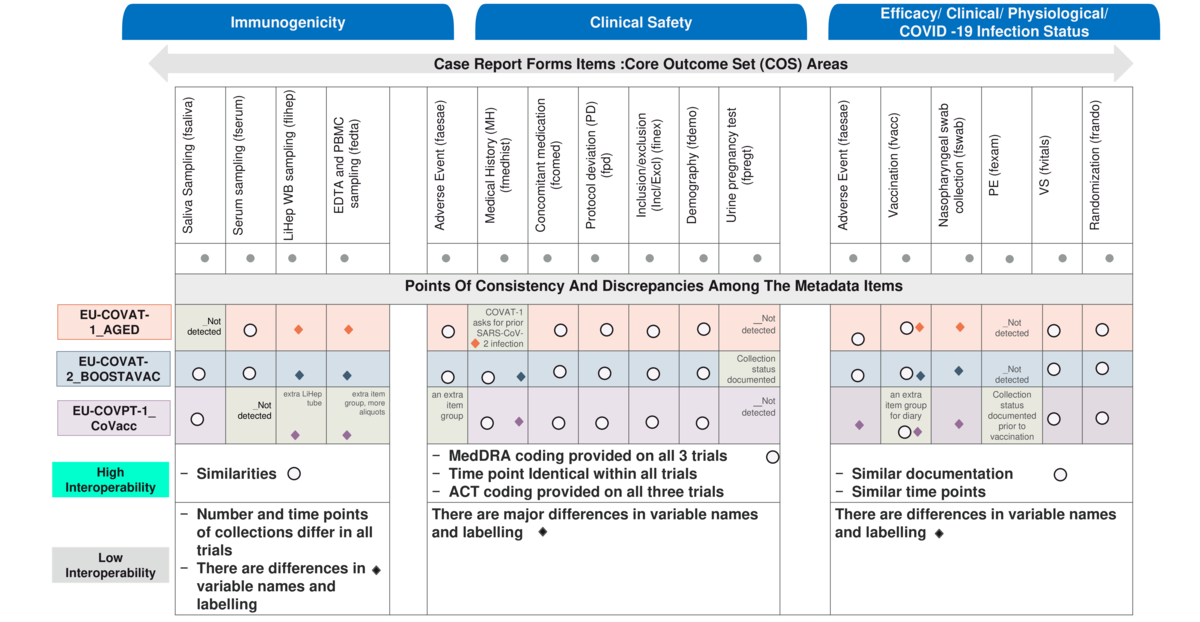
Metadata interoperability based on COS. EDTA: ethylenediaminetetraacetic acid; LiHep: lithium heparin; MedDRA: Medical Dictionary for Regulatory Activities; PBMC: peripheral blood mononuclear cell; PD: protocol deviation; PE: physical examination; VS: vital sign; WB: whole blood.

The points of consistency and discrepancies across the main core areas (eg, immunogenicity, safety, efficacy, clinical, and physiological aspects) among the metadata items of the 3 VACCELERATE clinical trials are presented in Tables 6-8 and Figure 5. The noncommonalities identified within the CRFs were attributed to differences in the 3 populations (aged 75+, adult, and pediatric), variations in protocol design, and differing vaccination patterns (Figures 3 and 5). Additionally, differences in the coding and labeling of variables, as well as in the item groups, were identified. These discrepancies required clarification to enhance interoperability.

**Table 6:** Points of consistency and discrepancies among the metadata items: core area IMMUNOGENICITY.

Case report form	EU-COVAT-1_AGED	EU-COVAT-2_BOOSTAVAC	EU-COVPT-1_CoVacc	Commonality and noncommonality
Saliva sampling (fsaliva)	Not detected	Collection status with time point and aliquot label before vaccination, on immune response evaluation, and on follow-up	Collection status with time point and aliquot label before vaccination and on follow-up	Identical for COVAT-2 and COVPT-1 Missing on COVAT-1
Serum sampling (fserum)	Collection status with time point and aliquot label before vaccination, on immune response evaluation, and on follow-up	Collection status with time point and aliquot label before vaccination, on immune response evaluation, and on follow-up	Not detected	Identical for COVAT-1 (form name differs fserumn) and COVAT-2 Missing on COVPT-1
LiHep^a^ WB^b^ sampling (flihep)	Collection status with time point and aliquot label before vaccination and on immune response evaluation	Collection status with time point and aliquot label before vaccination, on immune response evaluation, and on follow-up	Collection status with time point and aliquot label before vaccination and on follow-up	The number and time points of collections differ in all trials There are minor differences in variable names and labeling COVPT-1 has an extra LiHep tube
EDTA^c^ and PBMCs^d^ sampling (fedta)	EDTA collection status with time point and aliquot label before vaccination, on immune response evaluation, and on follow-up. PBMC only before vaccination	EDTA and PBMC collection status with time point and aliquot label before vaccination, on immune response evaluation, and on follow-up	EDTA collection status with time point and aliquot label before vaccination and on follow-up. PBMC for selected sites only on follow-up	The number and time points of collections differ in all trials There are differences in variable names and labeling COVPT-1 has an extra item group and more aliquots

^a^LiHep: lithium heparin.

^b^WB: whole blood.

^c^EDTA: ethylenediaminetetraacetic acid.

^d^PBMC: peripheral blood mononuclear cell.

**Table 7:** Points of consistency and discrepancies among the metadata items: core area CLINICAL SAFETY.

Case report form	EU-COVAT-1_AGED	EU-COVAT-2_BOOSTAVAC	EU-COVPT-1_CoVacc	Commonality and noncommonality
Adverse event (faesae)	Documentation forms available throughout the enclosure complemented by an optional unscheduled visit	Documentation forms available throughout the enclosure complemented by an optional unscheduled visit	Documentation forms available throughout the enclosure complemented by an optional unscheduled visit	Identical for COVAT-1 and COVAT-2 COVPT-1 has an extra item group MedDRAa coding provided on all three trials
MH^b^ (fmedhist)	MH documented before vaccination	MH documented before vaccination	MH documented before vaccination	Time points identical within all trials Major differences in variable names and labeling COVAT-1 asks for prior SARS-CoV-2 infection MedDRA coding provided on all 3 trials
Concomitant medication (fcomed)	Documentation forms available throughout the enclosure	Documentation forms available throughout the enclosure	Documentation forms available throughout the enclosure	Identical plus ATCc coding was provided on all 3 trials
PD^d^ (fpd)	PD documented on completion	PD documented on completion	PD documented on completion	Identical
Inclusion/exclusion (finex)	Inclusion/exclusion documented before enclosure	Inclusion/exclusion documented before enclosure	Inclusion/exclusion documented before enclosure	Identical
Demography (fdemo)	Demography documented before vaccination	Demography documented before vaccination	Demography documented before vaccination	Time points identical within all trials Differences in variable names and labeling COVAT-2 asks for race COVAT-1 and COVAT-2 for prior vaccination For COVPT-1, one inclusion criterion is not to be vaccinated before
Urine pregnancy test (fpregt)	Not detected	Collection status documented before vaccination and on an unscheduled visit	Not detected	N/Ae

^a^MedDRA: Medical Dictionary for Regulatory Activities.

^b^MH: medical history.

^c^ATC: Anatomical Therapeutic Chemical.

^d^PD: protocol deviation.

^e^N/A: not applicable.

**Table 8:** Points of consistency and discrepancies among the metadata items: core area EFFICACY/CLINICAL/PHYSIOLOGICAL/COVID INFECTION STATUS.

Case report form	EU-COVAT-1_AGED	EU-COVAT-2_BOOSTAVAC	EU-COVPT-1_CoVacc	Commonality and noncommonality
Patient diary (fdiary)	Diary collected on immune response evaluation	Diary collected on immune response evaluation	Diary administration on the second vaccination and first follow-up	Similar for COVAT-1 and COVAT-2 Difference to COVPT-1
Vaccination (fvacc)	Vaccination documented on baseline visit	Vaccination documented on time points based on the trial arm	Vaccination documented on time points based on the trial arm	Similar documentation There are differences in variable names and labeling COVPT-1 has an extra item group for the diary.
Nasopharyngeal swab collection (fswab)	Not detected	Not detected	Collection status documented before vaccination	N/A^a^
PE^b^ (fexam)	PE documented on screening, baseline, and immune response evaluation (in case of severe adverse event)	PE documented on screening, baseline, follow-up, and immune response evaluation	PE documented on screening, baseline, and follow-up	Similar documentation There are differences in variable names and labeling
VS^c^ (fvitals)	VS documented on screening, baseline, immune response evaluation, and in case of unscheduled visit	VS documented on baseline, follow-up, and in case of unscheduled visit	VS documented on screening and on any pre- and postvaccination visits	Similar time points Identical documentation
Randomization (frando)	Randomization documented on enrollment	Randomization documented on enrollment	Randomization documented on enrollment	Identical

^a^N/A: not applicable.

^b^PE: physical examination.

^c^VS: vital sign

### Interoperability in Data Management and Systems

VACCELERATE received a significant boost through the selection of the CTCC as the sole DM provider, with system database development coordinated by a single individual. This eliminated the need for coordination across multiple trial units. For the CTCC, the standardized format adopted within the VACCELERATE project utilized a data system featuring an installation of the Anju [16] (formerly OmniComm) TrialMaster clinical database management system, accompanied by metadata.

**Figure 6:**
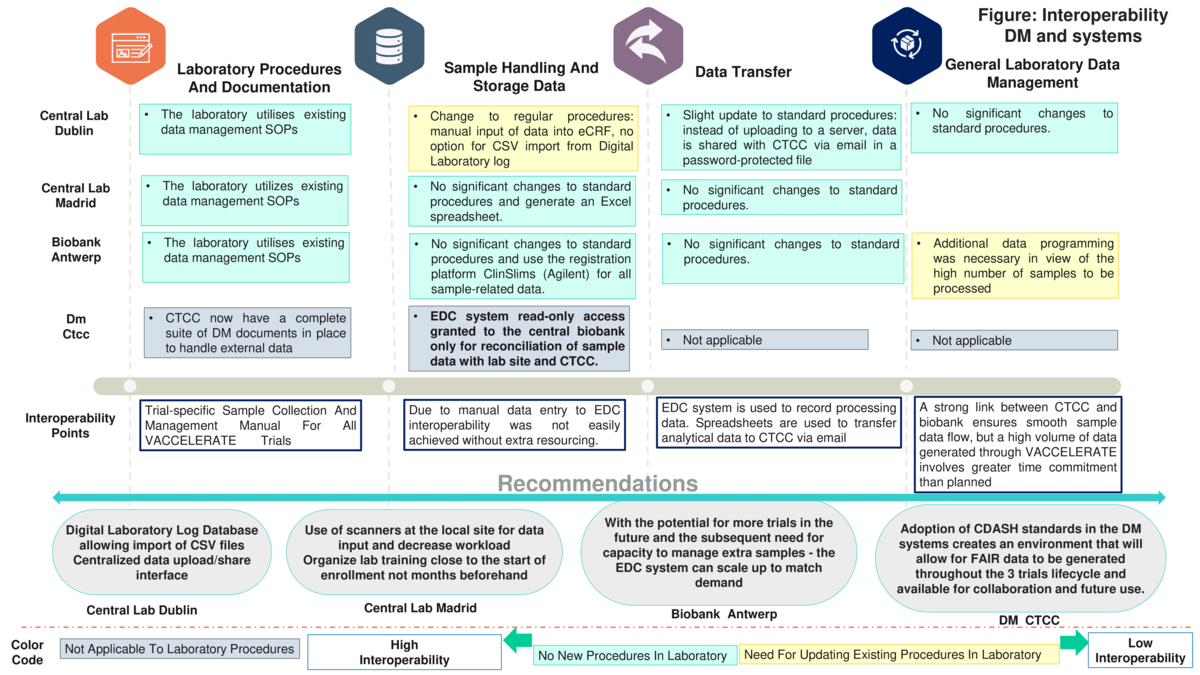
Interoperability between the data management and systems. CDASH: Clinical Data Acquisition Standards Harmonization; CTCC: Clinical Trials Centre Cologne; DM: data management; eCRF: electronic case report form; EDC: Electronic Data Capture; FAIR: Findable, Accessible, Interoperable, and Reusable; SOP: standard operating procedure.

Data transfer using tried-and-tested methods common to all sites (spreadsheet format) enabled a quick and relatively secure exchange, which proved highly effective. This process was further facilitated by the implementation of the TrialMaster system, which generated templates that could be adapted for use with spreadsheets. The responses received from the laboratories and CTCC are shown in Figures 3 and 6, while the questionnaire results are presented in Table S14 in Multimedia Appendix 2.

## Discussion

### Principal Findings

To the best of our knowledge, this is the first report to assess the degree of data commonality and interoperability between vaccine clinical trial protocols, the master protocol template, metadata, and data systems and management. In this study, we considered multiple parameters for the first time to enhance and improve interoperability across the developed COVID-19 vaccine clinical trial and master protocols, based on the selected COS areas: (1) immunogenicity, (2) safety, (3) efficacy/clinical/physiological outcomes, and (4) other outcomes of interest. This is significant because the lack of standardization in raw data processing is a well-known issue that exacerbates data inhomogeneity. This often results in errors and significant time delays, reducing the reusability and interoperability of data within protocols. To address this, the various protocol formats were homogenized by manually identifying discrepancies and commonalities among the selected parameters.

The database systems prepared by the CTCC already demonstrate substantial use of CDASH, a key enabler for the implementation of other CDISC standards. CDASH plays a crucial role in supporting the Study Data Tabulation Model (SDTM) [17]. Transforming study data to SDTM could also prove beneficial in the context of data sharing, especially as data interoperability becomes increasingly important. The adoption of CDASH standards [18] within the VACCELERATE DM systems establishes an environment conducive to generating FAIR data throughout the life cycle of the 3 trials. This approach ensures that the data will be available for collaboration and future use.

Pooling the metadata from the VACCELERATE studies would create a valuable metadata resource, independent of the underlying data. This resource could potentially be made accessible (eg, via the VACCELERATE official website and the ECRIN data-sharing repository for COVID-19 trials, EOSC-Life) [19], offering a unique opportunity for other sponsors to design related studies, particularly those utilizing the VACCELERATE infrastructure. The VACCELERATE metadata, as a shared resource, could play a pivotal role in standardizing data items and the controlled terminologies used within categorized questions, thus contributing to greater harmonization and interoperability in future research.

Education and training are critical for the successful implementation of FAIR DM in clinical data. Therefore, additional CDISC training for DM staff could be beneficial, both within VACCELERATE and more broadly, to support the adoption of data standards. Metadata harmonization should be completed before database development.

Although the DM staff at the CTCC are highly experienced and have already implemented international standards, there is a lack of planning for long-term data storage and reuse. This occurred because it was not anticipated during the database setup phase and due to the lack of a long-term data sharing budget, either linked to VACCELERATE itself or allocated to a data-sharing repository. Our report suggests that metadata harmonization planning should begin before database development and be supported by requirements (eg, CDASH) and oversight throughout the entire database setup phase, as well as any subsequent amendments. All subcontractors should be informed and included in this planning process. Furthermore, generating metadata and SDTM data for secondary use can be done under less time pressure compared with other aspects of standards implementation, such as the initial development of eCRFs. Conversely, while the final application of both is likely to occur at the end of the study, this does not mean the work must be confined to that time frame. Transformations can be planned and tested at any stage, using dummy data if necessary, as can the generation of metadata.

VACCELERATE highlights some of the challenges in making data standards a practical reality. At the same time, it provides opportunities to address these challenges, developing methods that can help embed data standards within noncommercial clinical research.

VACCELERATE received a substantial boost from the selection of the CTCC as the sole DM provider, with system database development coordinated by a single individual. This approach eliminated the need for coordination among different trial units, resulting in a high degree of uniformity within the system. The DM systems used in VACCELERATE are relatively new and have the potential to perform well with the limited number of sites currently using them. To effectively test these DM systems, it is necessary to have multiple sites utilizing them, with additional trials planned for later stages of the rollout. While the central Electronic Data Capture (EDC) system (TrialMaster) was used to manage sample processing data, there were concerns from the laboratory sites and the central biobank regarding the time required to enter sample data, resolve errors, and reconcile site datasets with the EDC. Transferring data using tried-and-tested methods common to all sites (spreadsheet format) enabled a harmonized, rapid, and relatively secure exchange. Before a scanning system was adopted, allowing sites to upload processing data directly into the EDC could significantly reduce the time constraints associated with manual data entry.

As only 3 clinical studies were funded under the VACCELERATE project, these results were not applied to any newly registered studies. However, as the objective of the VACCELERATE project has now been extended to other vaccine-preventable infectious diseases with pandemic potential, there will be a broader opportunity in the near future to implement the results presented here. Nevertheless, these trials provide valuable insights and a scalable framework for future vaccine studies, especially in the context of epidemics and pandemic preparedness. The methodology and protocols of these trials address critical questions regarding vaccine immunogenicity, reactogenicity, and optimal dosing strategies across diverse populations, including older adults (≥75 years), the general adult population, and pediatric patients. By using adaptive, multinational protocols, these trials simulate the real-world conditions of pandemic response, where speed, adaptability, and inclusivity are critical.

The methodologies from these trials are highly generalizable and provide a robust blueprint for future vaccine clinical studies within research networks. The inclusion of underrepresented populations, such as older adults and children, ensures that future vaccine trials will generate comprehensive, inclusive data applicable to all demographics. Additionally, the adaptive design of the protocols used in these studies allows for future modifications in response to emerging data and infectious diseases—a critical feature for addressing the dynamic nature of pandemics and evolving infectious threats.

Beyond the outcomes of individual trials, these studies contribute to the establishment and strengthening of international vaccine research networks. The collaborative, multicentric nature of the VACCELERATE network demonstrates the feasibility of harmonized vaccine research across borders, promoting shared protocols, centralized data analysis, and the dissemination of findings. This approach not only accelerates the vaccine development process but also strengthens global preparedness by creating investigator-initiated clinical research infrastructure and expertise that can be rapidly mobilized for emerging epidemics.

Moreover, the lessons learned from these trials can inform the design and implementation of studies for vaccines targeting other infectious diseases with pandemic potential, such as influenza or novel zoonotic pathogens. The emphasis on immunogenicity, dosing strategies, and booster efficacy can be adapted to various pathogens, ensuring that the evidence generated remains both relevant and actionable.

In summary, the VACCELERATE trials serve as a cornerstone for advancing and standardizing vaccine research methodologies. They underscore the importance of inclusivity, adaptability, and international collaboration, setting a precedent for future studies aimed at mitigating the impact of pandemics and safeguarding global public health.

### Limitations

This paper presents a novel approach to evaluating the degree of data commonality and interoperability between vaccine clinical trial protocols, the study master protocol template, metadata, and data systems and management. It did not benefit from preexisting methodologies, and its scope may therefore be limited by the specific cases examined. Nonetheless, it is anticipated to provide valuable insights that contribute to the improvement of clinical research data sharing and reuse.

### Conclusions

This study demonstrates that using master protocols can significantly enhance trial operational efficiency and data interoperability when similar infrastructure and DM procedures are consistently adopted across multiple trials. To achieve data interoperability while preserving meaning in interpretation and analysis, shared data should be structured, described, formatted, and stored using widely recognized data and metadata standards.

## Appendix

Multimedia Appendix 1 Additional analysis.

Multimedia Appendix 2 VACCELERATE central laboratories: Data Management Questionnaire.

## Abbreviations

AE: adverse event
CDASH: Clinical Data Acquisition Standards Harmonization
CDISC: Clinical Data Interchange Standards Consortium
CEPHR: Centre for Experimental Pathogen Host Research
COS: core outcome set
CRF: case report form
CTCC: Clinical Trials Centre Cologne
DB spec: Study Database Specification
DM: data management
eCRF: electronic case report form
EDC: Electronic Data Capture
FAIR: Findable, Accessible, Interoperable, and Reusable
GDPR: General Data Protection Regulation
ICH-GCP: International Conference on Harmonization for Good Clinical Practice
ISCIII: Instituto de Salud Carlos III
mRNA: messenger RNA
SAE: serious adverse event
SDTM: Study Data Tabulation Model
UCD: University College Dublin

## Data Availability

The datasets used and analyzed during this study are available from the corresponding author upon reasonable request.

## References

[1] Interoperability in healthcare. Healthcare Information and Management Systems Society.

[2] Wilkinson Mark D, Dumontier Michel, Aalbersberg I Jsbrand Jan, Appleton Gabrielle, Axton Myles, Baak Arie, Blomberg Niklas, Boiten Jan-Willem, da Silva Santos Luiz Bonino, Bourne Philip E, Bouwman Jildau, Brookes Anthony J, Clark Tim, Crosas Mercè, Dillo Ingrid, Dumon Olivier, Edmunds Scott, Evelo Chris T, Finkers Richard, Gonzalez-Beltran Alejandra, Gray Alasdair J G, Groth Paul, Goble Carole, Grethe Jeffrey S, Heringa Jaap, 't Hoen Peter A C, Hooft Rob, Kuhn Tobias, Kok Ruben, Kok Joost, Lusher Scott J, Martone Maryann E, Mons Albert, Packer Abel L, Persson Bengt, Rocca-Serra Philippe, Roos Marco, van Schaik Rene, Sansone Susanna-Assunta, Schultes Erik, Sengstag Thierry, Slater Ted, Strawn George, Swertz Morris A, Thompson Mark, van der Lei Johan, van Mulligen Erik, Velterop Jan, Waagmeester Andra, Wittenburg Peter, Wolstencroft Katherine, Zhao Jun, Mons Barend (2016). The FAIR Guiding Principles for scientific data management and stewardship. Sci Data.

[3] Ohmann Christian, Banzi Rita, Canham Steve, Battaglia Serena, Matei Mihaela, Ariyo Christopher, Becnel Lauren, Bierer Barbara, Bowers Sarion, Clivio Luca, Dias Monica, Druml Christiane, Faure Hélène, Fenner Martin, Galvez Jose, Ghersi Davina, Gluud Christian, Groves Trish, Houston Paul, Karam Ghassan, Kalra Dipak, Knowles Rachel L, Krleža-Jerić Karmela, Kubiak Christine, Kuchinke Wolfgang, Kush Rebecca, Lukkarinen Ari, Marques Pedro Silverio, Newbigging Andrew, O'Callaghan Jennifer, Ravaud Philippe, Schlünder Irene, Shanahan Daniel, Sitter Helmut, Spalding Dylan, Tudur-Smith Catrin, van Reusel Peter, van Veen Evert-Ben, Visser Gerben Rienk, Wilson Julia, Demotes-Mainard Jacques (2017). Sharing and reuse of individual participant data from clinical trials: principles and recommendations. BMJ Open.

[4] VACCELERATE - European Corona Vaccine Trial Accelerator platform. VACCELERATE.

[5] Salmanton-García Jon, Stewart FA, Heringer S, Koniordou M, Álvarez-Barco Elena, Argyropoulos CD, Themistocleous SC, Valle-Simón Paula, Spivak O, Součková Lenka, Merakou C, Amélia Mendonça Maria, Joanna Davis R, Maria Azzini A, Askling HH, Vene S, Van Damme P, Steinbach A, Shiamakkides G, Seidel D, Olesen OF, Noula E, Macken A, Luís Catarina, Leckler J, Launay O, Isitt C, Hellemans M, Frías-Iniesta Jesús, Di Marzo R, Carcas AJ, Boustras G, Borobia AM, Barta I, Albus K, Akova M, Ochando J, Cohen-Kandli M, Jane Cox R, Husa P, Jancoriene L, Mallon P, Marques L, Mellinghoff SC, Nauclér Pontus, Tacconelli E, Tóth Krisztina, Zaoutis TE, Zeitlinger M, Cornely OA, Pana Z (2022). VACCELERATE volunteer registry: a European study participant database to facilitate clinical trial enrolment. Vaccine.

[6] Salmanton-García J, Wipfler P, Valle-Simón P, Merakou C, Kopsidas I, Bethe U, Steinbach A, Spivak O, Součková L, Mendonça MA, Koniordou M, Hellemans M, Frías-Iniesta J, Davis RJ, Barta I, Azzini AM, Askling HH, Argyropoulos CD, Álvarez-Barco E, Akova M, Bonten MM, Cohen-Kandli M, Cox RJ, Flisiak R, Husa P, Jancoriene L, Koscalova A, Launay O, Lundgren J, Mallon P, Marques L, Nauclér P, Ochando J, Pana Z, Tacconelli E, Tóth K, Trelle S, van Damme P, Zaoutis TE, Zeitlinger M, Albus K, Stewart FA, Hofstraat SH, Bruijning-Verhagen P, Cornely OA (2023). VACCELERATE Site Network: Real-time definition of clinical study capacity in Europe. Vaccine.

[7] Argyropoulos CD, Leckler J, Salmanton-García Jon, Constantinou M, Alexandrou A, Themistocleous S, Noula E, Shiamakkides G, Nearchou A, Stewart FA, Albus K, Koniordou M, Kopsidas I, Spivak O, Hellemans M, Hendrickx G, Davis RJ, Azzini AM, Simon PV, Carcas-Sansuan AJ, Askling HH, Vene S, Prellezo JB, Álvarez-Barco Elena, Macken AJ, Di Marzo Romina, Luís Catarina, Olesen OF, Frias Iniesta Jesus A, Barta I, Tóth Krisztina, Akova M, Bonten MMJ, Cohen-Kandli M, Cox RJ, Součková Lenka, Husa P, Jancoriene L, Launay O, Lundgren J, Mallon P, Armeftis C, Marques L, Naucler P, Ochando J, Tacconelli E, van Damme Pierre, Zaoutis T, Hofstraat S, Bruijning-Verhagen P, Zeitlinger M, Cornely OA, Pana ZD (2023). Enhancing public health communication regarding vaccine trials: design and development of the Pan-European VACCELERATE toolkit. JMIR Public Health Surveill.

[8] Poulimeneas D, Koniordou M, Kousi D, Merakou C, Kopsidas I, Tsopela GC, Argyropoulos CD, Themistocleous SC, Shiamakkides G, Constantinou M, Alexandrou A, Noula E, Nearchou A, Salmanton-García Jon, Stewart FA, Heringer S, Albus K, Álvarez-Barco Elena, Macken A, Di Marzo R, Luis C, Valle-Simón Paula, Askling HH, Hellemans M, Spivak O, Davis RJ, Azzini AM, Barta I, Součková Lenka, Jancoriene L, Akova M, Mallon PWG, Olesen OF, Frias-Iniesta J, van Damme P, Tóth Krisztina, Cohen-Kandli M, Cox RJ, Husa P, Nauclér Pontus, Marques L, Ochando J, Tacconelli E, Zeitlinger M, Cornely OA, Pana ZD, Zaoutis TE, VACCELERATE Consortium (2023). The challenges of vaccine trial participation among underserved and hard-to-reach communities: an internal expert consultation of the VACCELERATE consortium. Vaccines (Basel).

[9] Clinical studies. VACCELERATE.

[10] Neuhann JM, Stemler J, Carcas A, Frías-Iniesta Jesús, Bethe U, Heringer S, Tischmann L, Zarrouk M, Cüppers Arnd, König Franz, Posch M, Cornely OA (2022). A multinational, phase 2, randomised, adaptive protocol to evaluate immunogenicity and reactogenicity of different COVID-19 vaccines in adults ≥75 already vaccinated against SARS-CoV-2 (EU-COVAT-1-AGED): a trial conducted within the VACCELERATE network. Trials.

[11] Salmanton-García Jon, Wipfler P, Leckler J, Nauclér Pontus, Mallon PW, Bruijning-Verhagen PC, Schmitt H, Bethe U, Olesen OF, Stewart FA, Albus K, Cornely OA, VACCELERATE Consortium (2024). Predicting the next pandemic: VACCELERATE ranking of the WorldHealth Organization's Blueprint forAction toPreventEpidemics. Travel Med Infect Dis.

[12] (2023). Clinical Data Interchange Standards Consortium - Clinical Data Acquisition Standards Harmonisation (CDISC CDASH). Clinical Data Interchange Standards Consortium.

[13] Clear data. Clear impact. Clinical Data Interchange Standards Consortium (CDISC).

[14] Soriano JB, Murthy S, Marshall JC, Relan P, Diaz JV, WHO Clinical Case Definition Working Group on Post-COVID-19 Condition (2022). A clinical case definition of post-COVID-19 condition by a Delphi consensus. Lancet Infect Dis.

[15] World Health Organization (WHO) (2021). A clinical case definition of post COVID-19 condition by a Delphi consensus. WHO.

[16] (2022). Anju Software.

[17] (2021). Clinical Data Interchange Standards Consortium - Study Data Tabulation Model (CDISC SDTM). Clinical Data Interchange Standards Consortium (CDISC).

[18] Standards. Clinical Data Interchange Standards Consortium.

[19] European Clinical Research Infrastructure Network.

