# Supplementary material for: Data Interoperability in COVID-19 Vaccine Trials: Methodological Approach in the VACCELERATE Project
Source: JMIR Med Inform. 2025 Mar 7;13:e65590. doi: 10.2196/65590 (PMC11928774; doi:10.2196/65590)
Supplement: Multimedia Appendix 1 [file medinform_v13i1e65590_app1.docx]

# Appendix A

Table1: Summary of the important aspects that contributed to the interoperability among the core outcome set (COS: a) Immunogenicity, b) Safety, c) Efficacy/clinical/physiological and, d) Other outcomes of interest

| **Comparison among the three specific clinical trial protocol** | **Comparison between the subprotocols and the predefined adult Master protocol** |
| --- | --- |
| - All three trial protocols are presented as adaptive, Phase II, Randomized, controlled, multicenter COVID-19 vaccine trials assessing in principle:  1. the immunogenicity 2. the safety profile (reactogenicity) of different vaccination strategies and age groups 3. Virus wild type and for the variants of concern (VOCs)  - The main safety endpoints as well as the follow up period (12 months) are identical for all three trials - The primary and secondary immunogenicity objectives (the short and long term humoral and cellular immune response) are assessed based on the same methodology/ laboratory assays | - The COS related to the immunogenicity profile of the vaccination strategies is reported in the three trial protocols, in an identical manner. - The same high level of data interoperability is observed for the second COS related to the definition and reporting of the safety profile. - On the contrary, the definition of the clinical and physiological COS are not included in the trial protocols, although there mentioned as part of the outcome trial dataset |

Table 2:Overview of the three VACCELERATE vaccine clinical trial protocols (2 adult and 1 paediatric trial).

| **Parameters** | **EU-COVAT-2_BOOSTAVAC**  **(Adult Trial)** | **EU-COVAT-1_AGED**  **(Adult trial)** | **EU-COVPT-1_CoVacc**  **(Paediatric Trial)** | **Similarities/Differences** |
| --- | --- | --- | --- | --- |
| **Title of the trial protocol**  EUDRACT | An International Multicentre, Phase 2, Randomised, Adaptive Protocol to determine the need for, optimal timing of and immunogenicity of administering a 4^th^ homologous mRNA vaccination dose against SARS-CoV2 in the general population (18+ years) already vaccinated against SARS-CoV-2 **(EU-COVAT-2_BOOSTAVAC).**  This trial is a sub-protocol embedded within the EU-COVAT master protocol.  EUDRA CT Nr: 2021-004889-35 | A multinational, Phase 2, Randomised, adaptive, protocol to evaluate immunogenicity and reactogenicity of different COVID-19 vaccines administration in older adults (≥75) already vaccinated against SARS-COV-2  **(EU-COVAT-1_AGED)**  This trial is a sub-protocol embedded within the EU-COVAT master protocol.  EUDRA CT Nr: 2021-004526-29 | Immunogenicity and reactogenicity of reduced COVID-19 mRNA vaccination regimen in children.  **(CoVacc)**  Paediatric Master protocol currently under review.  EUDRA CT Nr: 2021-005043-71 | Titles of all 3 subprotocols show that the focus lies on evaluating efficacy (immunogenicity) and safety (reactogenicity) of COVID-19 vaccines |
| **Trial Phase** | Phase II | Phase II | sub-Phase II | Identical |
| **Type of trial** | Randomised, controlled, adaptive, multicenter | Randomised, controlled, adaptive, multicenter | Randomised, comparative, multicenter (non-inferiority trial) | Identical |
| **Aim** | **Different timing** of administering a **4^th^ homologous mRNA vaccination dose** against SARS-CoV-2 in the **general population** to assess immunogenicity and safety | **Different booster strategies in individuals aged 75 years and older** already vaccinated against SARS-CoV-2 to assess immunogenicity and safety | **Single vs a two dose COVID-19** vaccination regimen in **paediatric subjects who are immunologically primed by natural infection** to assess immunogenicity and reactogenicity | **Similarities for adult trials**: both booster strategies, assess immunogenicity and reactogenicity.  **Difference**s: age range, timing  **Ped trial**: single vs two doses  **Identical to all three**: assess the immunogenicity and reactogenicity profile of COVID-19 vaccines. |

Table 3: Randomisation /cohorts/ blinding of the three VACCELERATE vaccine clinical trial protocols (2 adult and 1 paediatric trial).

| **Parameters** | **EU-COVAT-2**_**OOSTAVAC**  **(Adult Trial)** | **EU-COVAT-1_AGED**  **(Adult trial)** | **EU-COVPT-1_CoVacc**  **(Paediatric Trial)** | **Similarities/Differences** |
| --- | --- | --- | --- | --- |
| **Randomisation /cohorts/ blinding** | **Randomisation**: allocation will take place in the ratio 1:1:1: 1:1 across the control arm and the four intervention arms. Randomisation will be stratified by age, in the categories: 18-49; 50-59; ≥60  **Cohorts**: one control and 4 intervention arms  **Blinding**: No blinding is foreseen in this trial | Part A of the present trial in which individuals received a 3rd vaccination (first booster) is closed for further recruitment as of January 13, 2022. With the massive roll-out of booster campaigns throughout Europe, Part A was abandoned because of a poor recruitment rate.  **Part B.** Testing different vaccines as a 4th vaccination dose **(second booster)** for comparative assessment of their immunogenicity and safety against SARSCoV-2 wild-type and variants in the elderly  Allocation ratio of 1:1 BNT162b2 or mRNA-1273  **Group 1.** Individuals randomized to BNT162b2  **Group 2.** Individuals randomized to mRNA-1273  **Cohorts:** Six (12 arms)  **Blinding:** No blinding foreseen | The population consists of paediatric subjects with documented evidence of prior SARS-CoV-2 infection  The protocol will have two arms, a control arm and an intervention arm.  Subjects will be randomised to receive either  1)a 10 µg BNT162b2 first dose followed by a second 10 µg BNT162b2 dose  (control arm)  2) a single 10µg dose of BNT162b2 vaccine (intervention arm)  The visit schedule including planned assessments is detailed in Table 3. Subjects assigned to the control arm will receive their second vaccination 3-12 weeks, preferably 8 weeks after the first dose, as per local standard practice (Visit 3 D22-85) and will have 3 follow-up visits thereafter.  **Blinding:** No blinding foreseen | Reported in all trials the cohorts. And the randomisation process  **Blinding** is not foreseen in all 3 trials |

Table 4: Primary and secondary trial objectives of the three VACCELERATE vaccine clinical trial protocols (2 adult and 1 paediatric trial).

| **Parameters** | **EU-COVAT-2_BOOSTAVAC**  **(Adult Trial)** | **EU-COVAT-1_AGED**  **(Adult trial)** | **EU-COVPT-1_CoVacc**  **(Paediatric Trial)** | **Similarities/Differences** |
| --- | --- | --- | --- | --- |
| **Primary trial objective** | **Optimal timing of, and immunogenicity** of administering a **4^th^ homologous vaccination dose** against SARS-CoV-2 in the general population | **Reactogenicity between treatment** arms after a 4^th^ vaccination dose against SARS-CoV-2. | **Immune response** (geometric mean ratio of neutralizing titers) against wild type virus at day 28 | Similarities for Adult trials: both booster strategies, assess immunogenicity and reactogenicity  Differences: age range and timing.  Ped trial: single vs two doses  Identical to all three: assess the immunogenicity and reactogenicity profile of COVID-19 vaccines |
| **Secondary trial objectives** | To determine the **change in cellular immune response** measured by a direct from blood qPCR-based T-cell activation (dq-TACT) assay **at 14 days after 4^th^ dose vaccine** in comparison to prior to 4^th^ dose vaccination.  To correlate **humoral immune response, cellular immune responses and viral neutralising capacity** against **wild type SARS-CoV-2**  To determine the **durability of humoral and cellular immune responses after 3^rd^ dose of initial vaccination and shorter-term responses to 4^th^ dose** vaccination  To determine **rates of, and maximum disease severity** associated with confirmed SARS-CoV-2 infections occurring after enrolment in study participants before and after 4^th^ dose vaccine  To **explore primary and secondary endpoints stratified by incident infection pre-4^th^ dose vaccine or by any modification of vaccine epitope.** | **Ιmmunogenicity against wild-type SARS-CoV-2**  **Ιmmunogenicity against SARS-CoV-2 VOC**  l**ong-term humoral immune response**  (Reactogenicity and immunogenicity) | **Safety and reactogenicity of a single dose** COVID-19 vaccine regimen against SARS-CoV-2 in paediatric subjects  **Medium (6 months) and long term (12 month) humoral immune response** of the single COVID-19 vaccine dosing regimen against **wild-type SARS-CoV-2**.  **Short (28 days), medium and long term humoral immune response** of the single dose COVID-19 vaccine regimen against S**ARS-CoV-2 (VOCs)** | Similar secondary objectives assess the short- and long-term immune response till 12 months after the vaccine regimen against wild type and VOCs.  In 2/3 trials cellular immune response is added as exploratory endpoints |

Table 5: Safety, primary and secondary endpoints of the three VACCELERATE vaccine clinical trial protocols (2 adult and 1 paediatric trial).

| **Parameters** | **EU-COVAT-2 BOOSTAVAC**  **(Adult Trial)** | **EU-COVAT-1 AGED**  **(Adult trial)** | **EU-COVPT-1 CoVacc**  **(Paediatric Trial)** | **Similarities/**  **Differences** |
| --- | --- | --- | --- | --- |
| **Safety aim** | **Safety of different timing and 4^th^ booster strategies** in individuals vaccinated against SARS-CoV-2 | **Safety of a 4^th^ vaccination dose** against SARS-CoV-2 in the study population [older adults (≥75)] | **Safety of single vs a two dose COVID-19** vaccination regimen in **paediatric subjects who are immunologically primed by a natural infection** | Similar |
| **Primary endpoint** | **Increase in anti-RBD antibody titre to ≥500 IU/mL at day 14 post 4 th dose booster vaccine in those with anti-RBD antibody titre of ≤500 IU/mL immediately before 4 th dose booster vaccination**  or  **a 2-fold increase in anti-RBD antibody titre at day 14 following 4 th dose vaccination in those with anti-RBD titre of ≥500 IU/mL immediately before 4^th^.** | **2-fold antibody titre increases after a 4th vaccination** dose increase | **Geometric mean ratio of neutralizing titers against wild type virus** | Increase of antibody titre is similar in all trials.  Similar not identical primary endpoint between the adult trials due to particularities of the BOOSTAVAC trial |
| **Time point of the primary endpoint** | **14 days after 3^rd^ dose** | **14 days after the 4^th^ dose** | **at day 28** | Identical for the adult trials Similar for the ped trial |
| **Methodology/ Assay for the primary endpoint** | Quantitative enzyme-linked immunosorbent assay (Anti RBD-ELISA) | Quantitative enzyme-linked immunosorbent assay (Anti RBD-ELISA) | Virus Neutralization Assay | The difference is explained on what is reported on the protocol as primary and secondary endpoints |
| **Secondary endpoints -immunogenicity** | Antibody titre increase following 3rd dose measured by neutralising activity against wildtype virus (Virus Neutralisation Assay) in a subgroup 14 days after 3rd dose.  Immunogenicity against variants:  Change in neutralising capacity measured by neutralising activity against VOC; (Virus Neutralisation Assay) in a subgroup 14 days after 3^rd^ dose.  Long-term immunity:  Antibody titre level following 3^rd^ dose measured by a quantitative enzyme-linked immunosorbent assay (anti-RBD-ELISA assay).  Time-points will be defined in each sub-protocol under this master protocol.  Antibody titre level following 3rd dose measured by neutralising activity against wildtype virus (Virus Neutralisation Assay) in a subgroup. | Change in neutralizing antibody titre (Virus Neutralisation Assay) against wild-type 14 days after a 4th vaccination dose, to be performed in a subgroup only.  Immunogenicity against variants:  Change in neutralizing antibody titre (Virus Neutralisation Assay) against VOC 14 days after a 4^th^ vaccination dose, to be performed in a subgroup only.  Long-term immunity:  Antibody titre level 12 months after a 4^th^ vaccination dose measured by a quantitative enzyme linked immunosorbent assay (anti-RBD-ELISA assay).  Neutralizing antibody titre (Virus Neutralisation Assay) against wild-type SARS-CoV-2 at 12 months after a 4^th^ vaccination dose.  Neutralizing antibody titre (Virus Neutralisation Assay) against VOC at 12 months after a 4^th^ vaccination dose. | Safety and reactogenicity profile of each vaccine dose, and immunogenicity up to 12 months post-vaccination against wild-type virus and VOC. | See above |
| **Secondary endpoints**  **Safety** | **Unsolicited/Spontaneous AEs**/ until the end of trial  **Solicited AEs/** 7 days after 4^th^ dose  **Rate of serious adverse events (SAEs) Grade ≥3** according to the National Cancer Institute Common Toxicity Criteria **until up to three months** after 4 th dose or subject completes the study, whichever occurs first | **Unsolicited AEs /** until the end of trial  **Solicited AEs/**7 days after a 4^th^ dose  **Rate of SAEs**  Up to three months after 3rd dose **Grade ≥3** according to the National Cancer Institute Common Toxicity Criteria after a 4th vaccination dose | **Unsolicited AEs (local and systemic)** / for 14 days after each vaccine dose.  **Solicited systemic AEs** Grade ≥ 2 (AEs)/ 7 days after any vaccine dose, as measure of systemic reactogenicity  solicited local and systemic AEs for 7 days after each vaccine dose.  **Rate of SAEs Grade >3** on the Common Toxicity Criteria or Adverse Events of Special interest (AESI) until 12 months post-vaccination. | Identical duration for unsolicited AEs for the adult trials; follow up for the paediatric is 14 days  Identical  Identical |

Table 6: Inclusion and exclusion criteria, stratification/sub-populations and table of visit schedules of the three VACCELERATE vaccine clinical trial protocols (2 adult and 1 paediatric trial).

| **Parameters** | **EU-COVAT-2_BOOSTAVAC**  **(Adult Trial)** | **EU-COVAT-1_AGED**  **(Adult trial)** | **EU-COVPT-1_CoVacc**  **(Paediatric Trial)** | **Similarities/**  **Differences** |
| --- | --- | --- | --- | --- |
| **Inclusion criteria** | Already fully vaccinated (all-comers)  No contraindication against any of the vaccine products  Informed consent | Subject is ≥75 years old  Prior to study entry the subject was vaccinated with one of the following vaccination regimens (1st + 2nd + 3rd dose):  o BNT162b2 + BNT162b2 + BNT162b2  o BNT162b2 + BNT162b2 + mRNA-1273  o mRNA-1273 + mRNA-1273 + mRNA-1273  o mRNA-1273 + mRNA-1273 + BNT162b2  o ChAdOx-1-S + ChAdOx-1-S + BNT162b2  o ChAdOx-1-S + ChAdOx-1-S + mRNA-1273  The last dose of the above listed vaccinations must have been administered at least 1 month prior to study entry. Vaccination status should be documented in the source data and will be captured in the eCRF  Written informed consent from subject has been obtained. | Paediatric individuals (5 up to and including 11 years old) who are in good health or in a stable clinical condition and have obtained informed consent from their Legally Acceptable Representative (LAR), according to National Independent Ethic Committee requirements.  Inclusion criteria meet all of the following criteria:  (a) 5 up to and including 11 years of age on day of signing the informed consent form.  (b) In good health or stable clinical condition.  (c) Has documented evidence of a previous SARS-CoV-2 infection or presence of SARS-CoV-2 specific serum antibodies.  (d) LAR has reviewed the subject information and signed the informed consent form on behalf of the subject. | Differences observed among volunteers depending on trial participants age and other particularities of the trial. |
| **Stratification/sub-populations** | 1. **Age** (18-49; 50-59; 60-74; ≥75) 2. **Gender** 3. **Vaccine product used for primary vaccination** 4. NT162b2; mRNA1273; ChAdOx-1-S; other). 5. **Immune status**   (Competent, immunocompromised)  **Documented history of prior COVID-19 infection** (none, asymptomatic, symptomatic/pre-vaccination or post-vaccination). | Subgroup analyses will be performed by gender and documented history of prior COVID-19 infection (yes/no), respectively (page 67). | Stratification included as term but there is no additional information to add.  Descriptive subgroup analyses will be performed by sex. | Similar and based on the trial particularities (age groups), vaccine scheme etc. |
| **Table of visit schedules** | Yes | Yes | Yes | It is similar in all three trials with visits description |
| **Exclusion criteria** | 1. Participation in other interventional trials. 2. Use of drugs with significant interaction with the investigational product. 3. Diseases or findings that may have a significant effect on the target variables and which may therefore mask or inhibit the therapeutic effect under investigation. 4. Pregnant women and nursing mothers. 5. Persons with any kind of dependency on the principal investigator or employed by the sponsor or principal investigator. 6. Legally incapacitated persons. 7. Persons held in an institution by legal or official order | 1. Prior to study entry the subject got vaccinated with a regimen not included in the list given above. 2. Last anti-SARS-CoV-2 vaccine dose administered less than one month prior to study entry. 3. Vaccination against a disease other than COVID-19 within 2 weeks prior to study entry. Only exception: Influenza vaccination which is allowed at any time. 4. Subjects with any significant or uncontrolled disease posing a risk due to vaccination as judged by the investigator. 5. Current immunosuppressive therapy, for example continuous glucocorticosteroid treatment equivalent to >10 mg/day prednisolone. 6. Subject simultaneously participates in another clinical trials or has participated in the past 30 days. 7. Subjects unable to report solicited adverse events. 8. Subject with any contraindications to the vaccines in the trial. | 1. Has previously received any investigational or licensed COVID-19 vaccine. 2. Has known congenital or acquired immune disorder or immunodeficiency that may interfere with vaccine response e.g. known infection with human immunodeficiency virus (HIV) with low CD4 count or other immunosuppression at time of signing informed consent form. 3. Has a history of autoimmune disease or an active autoimmune disease requiring therapeutic intervention (including systemic glucocorticoids), or findings that may have a significant effect on the target endpoints and which may therefore mask or inhibit the therapeutic effect under investigation as judged by the investigator. 4. Bleeding diathesis or condition associated with prolonged bleeding that would, in the opinion of the investigator, contraindicate intramuscular injection or venepuncture. 5. History of severe adverse reaction associated with a vaccine and/or severe allergic reaction (e.g. anaphylaxis) to any component of the study intervention(s). 6. Receipt of medications intended to prevent COVID-19, in case of such medications receiving approval in this age group during the trial. 7. Uses drugs with significant interaction with the investigational product or has any contraindications as per the Summary of Product Characteristics. 8. Other medical or psychiatric condition or laboratory abnormality that may increase the risk of study participation or, in the investigator’s judgment, make the subject inappropriate for the study. 9. Has any kind of dependency on the principal investigator or member of the study team or LAR is employed by the sponsor or principal investigator. 10.Is unable to report solicited adverse events. | Differences according to the trial participants. |

Table 7: Remaining considered parameters of the three VACCELERATE vaccine clinical trial protocols (2 adult and 1 paediatric trial).

| **Parameters** | **EU-COVAT-2_BOOSTAVAC**  **(Adult Trial)** | **EU-COVAT-1_AGED**  **(Adult trial)** | **EU-COVPT-1_CoVacc**  **(Paediatric Trial)** | **Similarities/Differences** |
| --- | --- | --- | --- | --- |
| **IMP/dosage** | With marketing authorization at the time of each sub-protocol design  Dose approved by EMA and according to the respective summary of product characteristics (SPC). | An unexpected ADR is an ADR of which the nature or severity, outcome or frequency is not consistent with the applicable product information available for the IMP. ADRs listed in the Investigator’s Brochure or SPC, Information Sheet for Health Professionals are not regarded as unexpected. | Administration of the investigational product should be performed by an appropriately qualified, GCP-trained, and vaccine-experienced member of the study staff (e.g. physician, nurse, physician’s assistant, nurse practitioner, pharmacist, or medical assistant) as allowed by local, state, and institutional guidance. Investigational product should be administered intramuscularly into the deltoid muscle, preferably of the nondominant arm.  Standard vaccination practices must be adhered to including at least 15 minutes observation time and the vaccine must not be injected into blood vessels. Appropriate medication and other supportive measures for management of an acute hypersensitivity reaction should be available in accordance with local guidelines for standard immunization practices.  The licensed dose of BNT162b2 vaccine in children 5-11 years of age is 10μg contained in 0.2ml of the diluted vaccine. The diluent to be used is sodium chloride 9 mg/mL (0.9%) solution for injection. The standard schedule is two doses, a minimum of 21 days apart. | Reported, particularities and more explanation to the paediatric trial. |
| **Comparator/Control Group** | Vaccines to be administered in the control arms will be defined in each sub-protocol depending on the hypothesis and the booster strategies tested.  Subjects allocated to control arms may receive a homologous boost vaccine or not receive a boost dose. | No vaccination in the control group of the EU-COVAT subprotocol EudraCT no. 2021-004889-35, a separate sub-protocol embedded within the EUCOVAT master protocol (applies to Part A of the trial, in Part B this is an option only). | The protocol will have two arms, a control arm and an intervention arm. The population consists of paediatric subjects with documented evidence of prior SARS-CoV-2 infection. Subjects will be randomised to receive either 1) a 10 µg BNT162b2 first dose followed by a second 10 µg BNT162b2 dose (control arm), or 2) a single 10µg dose of BNT162b2 vaccine (intervention arm). | Reported according to the particularities of the trial. |
| **Duration of treatment and follow up of the study** | Subjects will receive only one booster dose of the vaccine.  Follow up of subject included will last for 12 months. | Treatment consists of a single 4th dose of each vaccine foreseen in this protocol. Follow up of subject included will last for 12 months. | Subjects assigned to the control arm will receive their second vaccination 3-12 weeks, preferably 8 weeks after the first dose, as per local standard practice (Visit 3 D22-85) and will have 3 follow-up visits thereafter. Subjects assigned to the intervention arm will also have 3 follow-up visits after their vaccination at visit 2.  Each paediatric subject will be followed for 1 year after completion of their vaccination regimen, i.e. after their last dose. | Visits are not identical between the adult and paediatric trial but the long term follow up of the adult and paediatric participants is at least 12 months. |
| **GCP/ethics** | In accordance with the valid versions of the trial protocol and the internationally recognised Good Clinical Practice Guidelines (ICH-GCP), including archiving of essential documents.  Consensus on ethical principles derived from international guidelines including the Declaration of Helsinki and Council for International Organizations of Medical Sciences (CIOMS) international ethical guidelines.  Applicable ICH-GCP guidelines dated July 1996 and its Addendum E6(R2) of June 2017.  Applicable laws and regulations. | The present trial will be conducted in accordance with the valid versions of the trial protocol and the ICH-GCP, including archiving of essential documents.  Consensus on ethical principles derived from international guidelines including the Declaration of Helsinki and CIOMS international ethical guidelines.  Applicable ICH-GCP guidelines dated July 1996 and its Addendum E6(R2) of June 2017.  Applicable laws and regulations. | The study will be performed in accordance with all applicable laws and regulations including the ICH-GCP the ethical principles that have their origins in the Declaration of Helsinki (current official version: Fortaleza, 2013; (5), the updated version of the General Data Protection Regulation (EU) 2016/679 (GDPR) and other applicable privacy laws. | Identical |
| **Financing** | Public funding – refer to specific sub-protocols for details of funding sources. | European Commission (EC) | Horizon 2020. Call: H2020-IBA-SC1-CORONAVIRUS-2020-4. Grant agreement number 101037867 | In all trials reported in similar way. |
| **Consent form** | Adult Participants | Adult Participants | Paediatric participants | Reported in all trials |
| **Insurance of patients** | All trial subjects enrolled are insured in accordance with regulatory requirements. The insurer's name, contact details, and policy number will be provided in the participant’s information sheet | All trial subjects enrolled are insured in accordance with regulatory requirements. The insurer's name, contact details, and policy number will be provided in the participant’s information sheet. | The sponsor certifies that it has taken out a liability insurance policy in accordance with national regulatory requirements. This insurance provides cover for damage to research subjects through injury or death caused by the study. The insurance of the Sponsor does not relieve the Investigator and the collaborators from maintaining their own liability insurance policy. | Reported in similar way in all 3 trials |
| **Sample size calculation** | **Sample size is preliminary and only the primary hypothesis testing is considered**  (see below for details) with the following assumptions:   1. Large sample normal approximation (score) test for a one-sample proportion (Stata Release 16.1) command: power one proportion) 2. Uninteresting (maximal) proportion of participants in a booster arm meeting the composite primary endpoint of 50%. 3. Proportion of participants in a booster arm meeting the composite primary endpoint of 69%. 4. Alpha (one-sided): 0.0125 5. Power: 90%   Using these assumptions, we calculated a sample size of 90 participants per arm (without dropouts, i.e., 100 with 10% dropouts). | **Sample size calculation with multiplicity adjustment within each cohort.**  When the sample size is 250 per randomized group (Group 1, Group 2) in Part B (275 without dropouts, i.e., assuming 8-10% dropouts), two-sided simultaneous 95% confidence intervals (with Bonferroni adjustment for 2 simultaneous confidence intervals within a cohort) for a proportion using the large sample normal approximation will extend no more than ±7.1% (percentage points) from the observed proportion. E.g., if the observed proportion is 50% (where the confidence interval is widest), the confidence interval ranges from about 42.9% to 57.1%. | The sample size calculation is based on the following assumptions:   1. The non-inferiority margin is 1.5-fold-difference between the Geometric Mean Titer (GMT) in the intervention and control arm or 0.176 absolute difference of GMT on log_10_ scale. 2. The standard deviation of neutralizing GMT on log_10_ scale we assumed a conservative estimate of 0.29 based on the current available data ([18]**)** Table 2) which is based on 5-11-year-old participants in the immunogenicity population of the immunobridging subset who had no serologic or virologic evidence of past or current SARS-CoV-2 infection up to the visit 1 month after the second dose and who had no history of COVID-19. Furthermore, we assumed that the standard errors are equal across groups. 3. The true difference of GMT on log_10_ scale is 0.05 | Reported in all 3 trials according to the particularities of each trial |
| **Data management** | Data management activities will be conducted by CTCC (Clinical Trials Centre Cologne). | Data management activities will be conducted by CTCC. | Data management activities will be conducted by CTCC. | Reported identical |

Table 8: Overview of the two adult VACCELERATE clinical trial protocols vs. the master protocol.

| **Parameter** | **EU-COVAT-2_BOOSTAVAC**  **(Adult trial)** | **EU-COVAT-1_AGED**  **(Adult trial)** | **EU-COVAT - Adult master protocol** | **Similarities/Differences** |
| --- | --- | --- | --- | --- |
| **Title of the trial**  **Master protocol**  **EUDRACT** | An International Multicentre, Phase 2, Randomised, Adaptive Protocol to determine the need for, optimal timing of and immunogenicity of administering a 4^th^ homologous mRNA vaccination dose against SARS-CoV2 in the general population (18+ years) already vaccinated against SARS-CoV-2 **(EU-COVAT-2 BOOSTAVAC).**  This trial is a sub-protocol embedded within the EU-COVAT master protocol.  **EUDRA CT Nr: 2021-004889-35** | A multinational, Phase 2, Randomised, adaptive, protocol to evaluate immunogenicity and reactogenicity of different COVID-19 vaccines administration in older adults (≥75) already vaccinated against SARS-COV-2  **(EU-COVAT-1_AGED)**  This trial is a sub-protocol embedded within the EU-COVAT master protocol.  **EUDRA CT Nr: 2021-004526-29** | A multinational, Phase 2, Randomised, Adaptive Master Protocol to Evaluate the Impact of Different COVID-19 Vaccine Booster Strategies in Adults Already Vaccinated Against SARS-CoV-2.  **(EU-COVAT)**  The Master Protocol has been drafted by consortia partners of the European vaccine trial network VACCELERATE  **EUDRA CT Nr**: N/A, specific to subprotocols | Both adult trial protocols comply to the main title information of the master protocol: to assess impact (immunogenicity and reactogenicity) of different vaccination booster strategies in adults. |
| **Phase of the trial** | Phase II | Phase II | Phase II | Same phase |
| **Type of the trial** | Randomised, controlled, adaptive, multicenter | Randomised, controlled, adaptive, multicenter | Randomised, adaptive, multicenter | Identical |
| **Aim** | **Different timing** of administering a **4^th^ homologous mRNA vaccination dose** against SARS-CoV-2 in the **general population** to assess immunogenicity and safety | **Different booster strategies in individuals aged 75 years and older** already vaccinated against SARS-CoV-2 to assess immunogenicity and safety | **Different booster strategies and timings** in order to assess their immunogenicity and safety against SARS-CoV-2 and its variant | Adult trials: booster strategies - similar, age range different |

Table 9: Randomisation /cohorts/ blinding of the two adult VACCELERATE clinical trial protocols vs. the master protocol.

| **Parameter** | **EU-COVAT-2_BOOSTAVAC**  **(Adult trial)** | **EU-COVAT-1_AGED**  **(Adult trial)** | **EU-COVAT - Adult master protocol** | **Similarities/Differences** |
| --- | --- | --- | --- | --- |
| **Randomisation /cohorts/ blinding** | **Randomisation**: allocation will take place in the ratio 1:1:1: 1:1 across the control arm and the four intervention arms. Randomisation will be stratified by age, in the categories: 18-49; 50-59; ≥60  **Blinding**: No blinding is foreseen in this trial | Part A of the present trial in which individuals received a 3rd vaccination (first booster) is closed to further recruitment as of January 13, 2022. With the massive roll-out of booster campaigns throughout Europe, Part A was abandoned because of a poor recruitment rate.  **Part B.** Testing of different vaccines as a 4th vaccination dose **(second booster)** for comparative assessment of their immunogenicity and safety against SARS-CoV-2 wild-type and variants in the elderly  Allocation ratio of 1:1 BNT162b2 or mRNA-1273  **Group 1.** Individuals randomized to BNT162b2  **Group 2.** Individuals randomized to mRNA-1273  **Cohorts:** Six (12 arms)  **Blinding:** No blinding foreseen | **Randomisation**: Subject fulfilling selection criteria will be randomised to the different treatment arms in the trial through a central procedure. Randomisation will be stratified according to: a) Vaccine product used for primary vaccination (detailed in the concerned sub-protocol), b) Trial Site, c) Gender, d) Immune status (competent, immunocompromised as applicable in concerned subprotocol), e) Documented history of prior COVID-19 infection (none, asymptomatic, symptomatic / pre-vaccination or post-vaccination).  The participants are randomised into the study arms as defined in each sub-protocol. Randomisation will be implemented by a 24/7-Internet service (ALEA 17.1, FormsVision BV, Abcoude, NL) and prepared centrally by the Institute of Medical Statistics and Computational Biology (IMSB) at the University of Cologne.  **Blinding:** Blinding procedures will be described as applicable in concerned subprotocols. | Reported in the Master protocol and in both adult trials, the considered subprotocols the cohorts and the randomisation process  **Blinding** is not foreseen in the adult trials |

Table 10: Primary and secondary trial objectives of the two adult VACCELERATE clinical trial protocols vs. the master protocol.

| **Parameter** | **EU-COVAT-2_BOOSTAVAC**  **(Adult trial)** | **EU-COVAT-1_AGED**  **(Adult trial)** | **EU-COVAT - Adult master protocol** | **Similarities/**  **Differences** |
| --- | --- | --- | --- | --- |
| **Primary trial objective** | **Optimal timing of, and immunogenicity** of administering a **4^th^ homologous vaccination dose** against SARS-CoV-2 in the general population | **Reactogenicity between treatment** arms after a 4^th^ vaccination dose against SARS-CoV-2. | **Immune response against wild-type SARS-CoV-2** of different booster strategies in individuals already fully vaccinated against SARS-CoV-2. | Similar primary objectives, the primary and secondary objectives/endpoints (including exploratory ones) as a whole core set of data are almost identical. |
| **Secondary trial objectives** | To determine the **change in cellular immune response** measured by a direct from blood qPCR-based T-cell activation (dq-TACT) assay **at 14 days after 4^th^ dose vaccine** in comparison to prior to 4^th^ dose vaccination.  To correlate **humoral immune response, cellular immune responses and viral neutralising capacity** against **wild type SARS-CoV-2**  To determine the **durability of humoral and cellular immune responses after 3rd dose of initial vaccination and shorter-term responses to 4th dose** vaccination  To determine **rates of, and maximum disease severity** associated with confirmed SARS-CoV-2 infections occurring after enrolment in study participants before and after 4^th^ dose vaccine  To **explore primary and secondary endpoints stratified by incident infection pre-4^th^ dose vaccine or by any modification of vaccine epitope.** | To compare **the immunogenicity against wild-type SARS-CoV-2 between treatment arms after a 4^th^ vaccination** dose against SARS-CoV-2.  To evaluate descriptively the **immunogenicity against SARS-CoV-2 VOC** between treatment arms after a 4^th^ vaccination dose against SARS-CoV-2.  To evaluate descriptively the **long-term humoral immune response** (reactogenicity and immunogenicity) of a 4^th^ vaccination dose against SARS-CoV-2. | Immune response against VOC of SARS-CoV-2. | Secondary objectives to assess the short- and long-term immune response till 12 months after the vaccine regimen against wild type and VOCs. |

Table 11: Safety, primary and secondary endpoints of the two adult VACCELERATE clinical trial protocols vs. the master protocol.

| **Parameter** | **EU-COVAT-2_BOOSTAVAC**  **(Adult trial)** | **EU-COVAT-1_AGED**  **(Adult trial)** | **EU-COVAT - Adult master protocol** | **Similarities/**  **Differences** |
| --- | --- | --- | --- | --- |
| **Safety** | Safety of different booster strategies in individuals vaccinated against SARS-CoV-2. | Safety of a 4^th^ vaccination dose against SARS-CoV-2 in the study population. | Safety of different booster strategies in individuals vaccinated against SARS-CoV-2. | Similar |
| **Exploratory endpoints** | - 1. The proportion of subjects reporting positive tests (PCR and/or antigen tests) for SARS-CoV-2 at each reporting time point after enrolment.   2. The proportion of subjects who receive 4^th^ dose vaccination at each specific timepoint that achieve a 2-fold increase in RBD antibody titre 14 days after the 4^th^ dose vaccine.   3. The proportion of subjects who receive 4th dose vaccination at each specific timepoint that achieve an anti-RBD antibody titre ≥500 IU/mL 14 days after the 4th dose vaccine.   4. Rate of 2-fold RBD antibody titre increase following 4^th^ dose vaccination measured by quantitative immunoassay targeting the spike 1 (S1) and nucleocapsid (NC) antibodies at 14 days after 4^th^ dose vaccine as compared to immediately before vaccination.   5. Analysis stratified by incident pre-4^th^ dose SARS-CoV-2 infections and any modification of vaccine epitope. | 1. Change in cellular immune response (CD4+ and CD8+ T cell response) measured by qPCR 14 days after 4th booster dose in a subgroup analysis. 2. Neutralizing antibody titre (Virus Neutralisation Assay) against newly emerging variants in bio-banked samples in a subgroup analysis after 4th vaccination dose. 3. Correlates of humoral immune response, cellular immune response and viral neutralising capacity against SARS-CoV-2 variants of concern (VOCs). | 1. Change in cellular immune response measured by qPCR 14 days after 3^rd^ dose in a subgroup analysis. 2. Neutralising capacity measured by neutralising activity against VOC in bio-banked samples in a subgroup analysis. | Similar primary objectives, the primary and secondary objectives/endpoints (including exploratory ones) as a whole core set of data are almost identical |
| **Secondary endpoints**  **safety** | **Unsolicited AEs**/ until the end of trial  **Solicited** **AEs**/ 7 days after 3^rd^ dose  Grade mild, moderate and severe according to CTCAE grading 1 to 3  **Rate of SAEs** Up to three months after 3^rd^ dose. Grade ≥3 according to the National Cancer Institute Common Toxicity Criteria | **Unsolicited AEs /** until the end of trial  **Solicited AEs/** 7 days after a 4^th^ dose  **Rate of SAEs** Up to three months after 3^rd^ dose. Grade ≥3 according to the National Cancer Institute Common Toxicity Criteria after a 4^th^ vaccination dose | **Unsolicited AEs** /until the end of trial.  **Solicited AEs/** for 7 days after 3^rd^ dose.  **Rate of SAEs** Grade ≥3 according to the National Cancer Institute Common Toxicity Criteria until up to three months after 3^rd^ dose. | Identical duration for unsolicited AEs for the adult trials follow up for the paediatric is 14 days  Identical  Identical |

**Table 12: Inclusion and exclusion criteria, stratification/sub-populations and table of visit schedules of the two adult VACCELERATE clinical trial protocols vs. the master protocol.**

| **Parameter** | **EU-COVAT 2_BOOSTAVAC**  **(Adult trial)** | **EU-COVAT-1_AGED**  **(Adult trial)** | **EU-COVAT - Adult master protocol** | **Similarities/Differences** |
| --- | --- | --- | --- | --- |
| **Inclusion criteria** | Already fully vaccinated adults (all-comers)  No contraindication against any of the vaccine products  Informed consent | Subject is ≥75 years old  Prior to study entry the subject was vaccinated with one of the following vaccination regimens (1st + 2nd + 3rd dose):  o BNT162b2 + BNT162b2 + BNT162b2  o BNT162b2 + BNT162b2 + mRNA-1273  o mRNA-1273 + mRNA-1273 + mRNA-1273  o mRNA-1273 + mRNA-1273 + BNT162b2  o ChAdOx-1-S + ChAdOx-1-S + BNT162b2  o ChAdOx-1-S + ChAdOx-1-S + mRNA-1273  The last dose of the above listed vaccinations must have been administered at least 1 month prior to study entry. Vaccination status should be documented in the source data and will be captured in the eCRF  Written informed consent from subject has been obtained. | Already fully vaccinated adults (all-comers, i.e. all that have no contraindication against any of the vaccines at time of enrolment).  No contra-indication against any of the vaccine products in the trial.  Informed consent. | Differences according to the trial participants |
| **Stratification/**  **sub-populations** | 1. **Age** (18-49; 50-59; 60-74; ≥75) 2. **Gender** 3. **Vaccine product used for primary vaccination** (B NT162b2; mRNA1273; ChAdOx-1-S; other) 4. **Immune status** (Competent, immunocompromised) 5. **Documented history of prior COVID-19 infection** (none, asymptomatic, symptomatic/pre-vaccination or post-vaccination) | Subgroup analyses will be performed by gender and documented history of prior COVID-19 infection (yes/no), respectively | 1. **Age (18-49; 50-59; 60-74; ≥75).** 2. **Gender** 3. **Vaccine product used for primary vaccination** (BNT162b2; mRNA-1273; ChAdOx-1-S; other). 4. **Immune status** (competent, immunocompromised) 5. **Documented history of prior COVID-19 infection** (none, asymptomatic, symptomatic/pre-vaccination or post-vaccination). | Identical for adult trials and Master protocol |
| **Table of visit schedules** | Yes | Yes | No |  |
| **Exclusion criteria** | 1. Participation in other interventional trials. 2. Use of drugs with significant interaction with the investigational product. 3. Diseases or findings that may have a significant effect on the target variables and which may therefore mask or inhibit the therapeutic effect under investigation. 4. Pregnant women and nursing mothers. 5. Persons with any kind of dependency on the principal investigator or employed by the sponsor or principal investigator. 6. Legally incapacitated persons. 7. Persons held in an institution by legal or official order | 1. Prior to study entry the subject got vaccinated with a regimen not included in the list given above. 2. Last anti-SARS-CoV-2 vaccine dose administered less than one month prior to study entry. 3. Vaccination against a disease other than COVID-19 within 2 weeks prior to study entry. Only exception: Influenza vaccination which is allowed at any time. 4. Subjects with any significant or uncontrolled disease posing a risk due to vaccination as judged by the investigator. 5. Current immunosuppressive therapy, for example continuous glucocorticosteroid treatment equivalent to >10 mg/day prednisolone. 6. Subject simultaneously participates in another clinical trials or has participated in the past 30 days. 7. Subjects unable to report solicited adverse events. 8. Subject with any contraindications to the vaccines in the trial. | 1. Participation in other interventional trials. 2. Use of drugs with significant interaction with the investigational product. 3. Diseases or findings that may have a significant effect on the target variables and which may therefore mask or inhibit the therapeutic effect under investigation. 4. Pregnant women and nursing mothers. 5. Persons with any kind of dependency on the principal investigator or employed by the sponsor or principal investigator. 6. Legally incapacitated persons. 7. Persons held in an institution by legal or official order. | Differences according to the trial participants |

Table 13: Remaining considered parameters of the two adult VACCELERATE clinical trial protocols vs. the master protocol.

| **Parameter** | **EU-COVAT-2_BOOSTAVAC**  **(Adult trial)** | **EU-COVAT-1_AGED**  **(Adult trial)** | **EU-COVAT - Adult master protocol** | **Similarities/Differences** |
| --- | --- | --- | --- | --- |
| **IMP/**  **dosage** | With marketing authorization at the time of each sub-protocol design  Dose approved by EMA and according to the respective summary of product characteristics (SPC) | An unexpected ADR is an ADR of which the nature or severity, outcome or frequency is not consistent with the applicable product information available for the IMP. ADRs listed in the Investigator’s Brochure or SPC, Information Sheet for Health Professionals, are not regarded as unexpected. | Dose to be administered and method of administration will be those approved by EMA and according to the respective summary of product characteristics. | Identical |
| **Comparator**  **/Control Group** | Vaccines to be administered in the control arms will be defined in each sub-protocol depending on the hypothesis and the booster strategies tested.  Subjects allocated to control arms may receive a homologous boost vaccine or not receive a boost dose. | No vaccination in the control group of the EU-COVAT subprotocol EudraCT no. 2021-004889-35, a separate sub-protocol embedded within the EUCOVAT master protocol (applies to Part A of the trial, in Part B this is an option only). | Vaccines to be administered in the control arms will be defined in each sub-protocol depending on the hypothesis and the booster strategies tested. Subjects allocated to control arms may receive a homologous boost vaccine or not receive a boost dose. | Reported to the Master protocol and adopted according to the objectives of the adult trials respectively. |
| **Duration of treatment and follow up of the study** | Subjects will receive only one booster dose of the vaccine | Treatment consists of a single 4^th^ dose of each vaccine foreseen in this protocol. Follow up of subject included will last for 12 months. | Subjects will receive only one booster dose of the vaccine. | Identical for adult trials and Master protocol (up to 12months) |
| **GCP/**  **ethics** | In accordance with the valid versions of the trial protocol and the ICH-GCP, including archiving of essential documents.  Consensus on ethical principles derived from international guidelines including the Declaration of Helsinki and CIOMS international ethical guidelines.  Applicable ICH-GCP guidelines dated July 1996 and its Addendum E6(R2) of June 2017.  Applicable laws and regulations. | The present trial will be conducted in accordance with the valid versions of the trial protocol and the ICH-GCP, including archiving of essential documents.  Consensus on ethical principles derived from international guidelines including the Declaration of Helsinki and CIOMS international ethical guidelines.  Applicable ICH-GCP guidelines dated July 1996 and its Addendum E6(R2) of June 2017.  Applicable laws and regulations. | The present trial will be conducted in accordance with the valid versions of the trial protocol and the ICH-GCP, including archiving of essential documents  Consensus on ethical principles derived from international guidelines including the Declaration of Helsinki and CIOMS international ethical guidelines.  Applicable ICH-GCP guidelines dated July 1996 and its Addendum E6(R2) of June 2017.  Applicable laws and regulations. | Identical |
| **Financing** | Public funding – refer to specific sub-protocols for details of funding sources. | European Commission | Public funding – refer to specific sub-protocols for details of funding sources. | Reported in all protocol and trials |
| **Consent form** | Adult Participants | Adult Participants | Adult Participants | Identical |
| **Insurance of patients** | All trial subjects enrolled are insured in accordance with regulatory requirements. The insurer's name, contact details, and policy number will be provided in the participant’s information sheet | All trial subjects enrolled are insured in accordance with regulatory requirements. The insurer's name, contact details, and policy number will be provided in the participant’s information sheet. | All trial subjects enrolled are insured in accordance with regulatory requirements. The insurer's name, contact details, and policy number will be provided in the participant’s information sheet. | Identical |
| **Sample size calculation** | **Sample size is preliminary and only the primary hypothesis testing is considered** (see below for details) with the following assumptions:   - 1. Large sample normal approximation (score) test for a one-sample proportion (Stata (Release 16.1) command: power one proportion)   2. Uninteresting (maximal) proportion of participants in a booster arm meeting the composite primary endpoint of 50%   3. Proportion of participants in a booster arm meeting the composite primary endpoint of 69%   4. Alpha (one-sided): 0.0125   5. Power: 90%   Using these assumptions, we calculated a sample size of 90 participants per arm (without dropouts, i.e., 100 with 10% dropouts). | **Sample size calculation with multiplicity adjustment within each cohort.**  When the sample size is 250 per randomized group (Group 1, Group 2) in Part B (275 without dropouts, i.e., assuming 8-10% dropouts), two-sided simultaneous 95% confidence intervals (with Bonferroni adjustment for 2 simultaneous confidence intervals within a cohort) for a proportion using the large sample normal approximation will extend no more than ±7.1% (percentage points) from the observed proportion. E.g., if the observed proportion is 50% (where the confidence interval is widest), the confidence interval ranges from about 42.9% to 57.1%. | Please refer to respective sub-protocols under this master protocol. | Reported in the Master protocol and adopted according to the objectives of the adult trials respectively. |
| **Coordination**  **Data management** | Data management activities will be conducted by CTCC | Data management activities will be conducted by CTCC | Data management activities will be conducted by CTCC | Identical |

**Table 14: Laboratory Questionnaire Results**

| **Measure** | **Central Lab – Dublin (UCD)** | **Central Lab – Madrid (ISCIII)** | **Biobank – Antwerp** | **DM – CTCC** | **Interoperability points** |
| --- | --- | --- | --- | --- | --- |
| **Laboratory Procedures and Documentation** | | | | | |
| **Lab Function** | Measure IgG levels  anti-RBD, anti-N  and neutralising antibody analysis | Cellular immunity measured by PCR | 1. Sample kit preparation and shipment 2. Label printing 3. Lab manual writing 4. Training on sample collection and processing 5. Shipment of specimen 6. Biobanking 7. Analytics. | N/A | N/A |
| **Lab DM SOPs** | Yes | Yes | Yes | N/A | All lab sites employ a trial specific **Sample Collection and Management Manual** for 3 trials |
| **DM SOPs reviewed** | Unaware of any feedback regarding the data management from CTCC to UCD. | No | No | Yes, CTCC now have a complete suite of DM documents in place to handle ‘external data’ | All lab sites involved in development and sign-off of Sample Collection and Management Manual. CTCC external DMPs and quality management documents developed in-house |
| **Data Dictionary in place** | Yes | No | Yes, Extra data for CoVacc since we will analyse SARS-CoV-2 positive samples in this study | Yes | Data dictionary accepted by sites |
| **Data Dictionary reviewed** | Yes |  | Yes | Yes | Data dictionary forms the basis of external data reconciliation file generated in TrialMaster (EDC system) |
| **DMP review** | No |  | No | Yes | DMPs were developed by CTCC and individual trial sponsors and did not involve labs.  *Note: The details reg. Lab are described in the External Data Reconciliation Plans. DMP does not cover this topic.* |
| **DMP development** | No |  | No | Yes | Labs not involved in developing DMPs |
| **Measure** | **Central Lab – Dublin (UCD)** | **Central Lab – Madrid (ISCIII)** | **Biobank – Antwerp** | **DM – CTCC** | **Interoperability points** |
| **Sample Handling and Storage Data** | | | | | |
| **Change handling normal practice** | Yes, the eCRF/database doesn’t allow the import of CSV files, it only accepts manual input, therefore, we had to dedicate additional personnel to manually input laboratory information to the database, while in other trials, this was done by importing a CSV template directly into the database. | No | No | No | With the use of EDC system with restricted access to handle lab processing data interoperability was not easily achieved without extra resourcing. |
| **Data from samples received** | They are recorded in paper format, with a form that comes with the sample kit. This information is then manually inputted into the CTCC eCRF/Database. However, here in UCD, besides keeping the paper records, we also have a Digital Laboratory log, where we keep all the information digitally, and we expand on the details about the samples processed (such as Scanning the Unique QR code of the sample, or cell count, etc). | Generate an excel spreadsheet | We use the registration platform ClinSlims (Agilent) for all sample related data (label printing, registration, reconciliation, shipments to other partners) | N/A | All lab sites use spreadsheet to record sample data |
| **Sample**  **received on database** | The data associated to the samples is recorded both in paper and digital form, and then, inputted manually to the eCRF by appropriately trained and certified research Staff. | N/A - Do not use the trial eCRF | NA since we can only make queries into the eCRF. | N/A | Manual data entry to EDC system |
| **Reconcile database queries** | Most of the reconciliation is done by the Central Laboratory in Antwerp, they contrast/compare the information on the tubes/samples, with the information provided by the CTCC (eCRF completed by each site), however, since we are a processing site, we received samples from Antwerp, alongside a digital shipping manifest, we also do a reconciliation of that digital manifest (info coming from CTCC and Antwerp) with the samples that we received here in the laboratory.  Once we make sure that there are no non-conformities, we sent a signed acknowledgement of the samples received, and we proceed with the analysis. | N/A - Do not use the trial eCRF | If non-conformities, we make queries into the eCRF that need to be solved by the local site. | EDC system read only access granted to central biobank for reconciliation as well as sample reconciliation data with lab site and CTCC. Automatic and manual edit checks for CRF reporting | High degree of interoperability with checks in place to resolve sample data queries. CTCC use central lab and the lab site to reconcile queries if they exist. |
| **Comment on sample handling data** | 1. Migrate from a paper base laboratory log to a digital laboratory log that could be stored locally, and subsequently shared with CTCC for a better oversight of the data collected at each site. 2. Create a template (CSV file) that could be used to import data into the database, rather than individually/manually inputting data into the Database. 3. Create a file sharing interface/server, where each site can upload the information required for the analysis (such as, results from IgG measurement or neutralizing capacity) | No | Use of scanners at local site would improve correct data input and decrease workload at local site and at central lab | With the potential for more trials in the future and subsequent need for capacity to manage extra samples - EDC system can scale up to match demand | Lab sites see greater opportunity to streamline data handling arrangements for the sample processing data as well as the analytical data that is generated. |
| **Measure** | **Central Lab – Dublin (UCD)** | **Central Lab – Madrid (ISCIII)** | **Biobank – Antwerp** | **DM – CTCC** | **Interoperability points** |
| **Data Transfer** | | | | | |
| **Change transfer normal practice** | Yes, Data resulting from laboratory assays, is usually uploaded to a file sharing website/server for other clinical trials. In this case (VACCELERATE), is sent by email. But other than that, the data requested, and format is very similar to other clinical trials | No | No | N/A | While the EDC system is used to record processing data, spreadsheets are used to transfer analytical data to CTCC via email |
| **Data transfer agreement** | Yes | No | Yes | Yes | Transfer agreements are in place that ensure that data is reconciled to the agreed format, the timing of data coincide with study milestones and complete. |
| **Analytic data storage prior transfer** | Excel files hosted in local servers. | Excel files datasheet | FASTA, excel output (only CoVacc) | N/A | All lab sites use spreadsheets to record analytical data |
| **Analytic data transfer to CTCC** | By email, on a password protected document. The password is sent either by regular Post, or by a separate email to the CTCC | Encrypted excel datasheet | format described in dictionary (would be an excel). Number of transfers still TBD | Predefined spreadsheet template is provided to the lab site for use in transferring data to CTCC | All lab sites use spreadsheets to transfer analytical data |
| **Analytic data reconcile queries** | Via email, we have direct contact with the CTCC, and if any discrepancy is found, we create a query log, where each of the non-conformities is address, and if necessary, the Central Biobank is consulted (Antwerp) | Via email and teleconference | TBD for CoVacc | CTCC take the lead in identifying missing sample data points in trial EDC and local lab database by comparing and then resolve differences | All sites have a standard reconciliation procedure to identify and resolve queries, good overlap occurs |
| **Analytic data transfer to Biobank** | Data is usually transfer to CTCC first, and they will provide the Central Biobank with any data that they are requiring.  However, the Central Biobank has created templates and a very detailed procedure to transfer samples (and shipment data) to the Central Biobank, but this wouldn’t be classified as “Analytics’’, as in, results from an assay, it would be mostly shipment data. |  | The central biobank does not receive results from the analyses done by the central research labs | N/A | Does not apply here as all data go to CTCC |
| **Biobank storage prior transfer** | N/A | N/A | electronically within the registration platform ClinSlims | N/A | High degree of interoperability with well-defined data sample collection and management manual in place |
| **Biobank data transfer to CTCC** | N/A | N/A | excel output password protected | N/A | Link between central biobank and CTCC well established with biobank acting as intermediary between CTCC and central labs to ensure standards are adhered to in data transfer |
| **CTCC data transfer to Biobank** | N/A | N/A | electronic link, password protected before download is enabled, zip-file containing excel files | CTCC sends biweekly listing of sample data to Biobank Antwerp for data cleaning and reconciliation in excel format | As above |
| **Biobank data reconcile with CTCC** | N/A | N/A | the central biobanking lab sends 2 excel files to CTCC containing all aliquots that were shipped to the 2 research labs (Dublin and Madrid) after each shipment. CTCC checks with the database and returns a query in case of non-conformities. The central biobanking laboratory should solve the query. | CTCC allows Biobank Antwerp access to EDC system to raise queries on sample data received from sites.  Bi-weekly listing from EDC system of sample data for reconciliation sent to  Biobank Antwerp with continuous checking of VAC1 codes throughout process | High degree of interoperability between Biobank Antwerp and CTCC due to the high volume of contact between the two sites refining transfer processes |
| **Comment on data transfer** |  |  | No |  |  |
| **General Laboratory Data Management** | | | | | |
| **DM practice with VACCELERATE** | No |  | Yes, Additional data programming was necessary in view of high numbers of samples to be collected and the exceptional high amounts of aliquots to be processed | N/A | A strong link exists between the CTCC and biobank to ensure the flow of sample data, from the lab sites to the biobank and from the biobank back to the sites, runs smoothly. |
| **DM recommendations** | 1. Digital Laboratory Log 2. Database that allows to import CSV files 3. Laboratory aliquots with 1 identifier only, that is both readable and scannable. At the moment, we have 2 identifiers in the sample label, a written code (Starts with the “VAC’’), which is not scannable, and a QR Code which is not written on the label, and has no similarity to the VAC, but it’s the only scannable piece of information in the sample tube. The question would be, why not have only 1 code (let’s say, the unique VAC code) that is both readable and scannable 4. Established a centralized data upload/share interface, with username and password to access it, where each lab can input their experimental data. |  | 1. There are a lot of problems with sample reconciliation due to numerous typos at the local site level due to carelessness. Use of scanners at local site for sample/aliquot registration, confirmation of shipment in sample shipment manifest 2. Pop-up error when saving the TrailMaster notes when there is a typo detected (based on preprogramed aliquot IDs), possibility to block copy/paste when completing the aliquot codes into the eCRF, double data input, allow possibility of a 2nd person to check the data before saving in TrialMaster 3. Discuss upfront possibilities to complete the eCRF with a minimum of possible errors (e.g. use of scanners at local sites for sample/aliquot registration and for confirmation of sample shipment manifest, use of list containing possible aliquot numbers, block copy/paste, …) 4. Keep the number of aliquots to be prepared per visit reasonable e.g. not 20 EDTA aliquots unless they will be used for sure 5. Organise lab training as close as possible to the actual start of enrolment and not months beforehand | Data management and systems provided by a single provider allows for a cohesive solution to the requirements of multi-site pan - European trial network.  With the potential to scale up more sites and vaccine trials, it will involve more sample processing data to manage.  The CTCC can absorb the extra workload through the DM structures put in place and the resources available. | Central Labs In agreement with the experience of Biobank Antwerp in that the high volume of data generated through VACCELERATE involves greater time commitment than planned.  The central lab and biobank agree that a time saving solution is needed to manage the processing of sample data. This could involve a rationalising of the sample identifier process (from 2 codes to 1) and digitally scan the identifier into the EDC system as opposed to manually entering data which causes excessive entry errors.  Coupled with a streamlined and digitised process to capture sample data in the EDC system would greatly reduce the need for data entry error checking, relevant training for site staff at the right time is key to successful implementation of site engagement with EDC systems |
