# Supplementary material for: Data Interoperability in COVID-19 Vaccine Trials: Methodological Approach in the VACCELERATE Project
Source: JMIR Med Inform. 2025 Mar 7;13:e65590. doi: 10.2196/65590 (PMC11928774; doi:10.2196/65590)
Supplement: Multimedia Appendix 2 [file medinform_v13i1e65590_app2.docx]

# Appendix B:

# VACCELERATE Central Laboratoires - Data Management Questionnaire.

| **1. Laboratory Procedures and Documentation** | |
| --- | --- |
| What central laboratory function do you perform for the VACCELERATE network? | Please list: |
| Did your laboratory have data management SOPs in place before you became involved with the VACCELERATE network? | Yes , No  If yes, please list appropriate SOP |
| Were your data management SOPs reviewed by Vaccelerate central organisation unit i.e., CTCC before the trials began? | Yes , No  If yes, describe briefly |
| Do you have a data dictionary for the VACCELERATE Laboratory sample data? | Yes , No |
| If yes, is it the same data dictionary for the 3 Vaccelerate protocols, i.e., CoVacc, BOOSTAVAC, Aged? | Yes , No |
| Was the data dictionary reviewed by CTCC prior to first data transfer? | Yes , No |
| Did you review the Vaccelerate Clinical Trial Data Management Plans that were developed for the 3 trials? | Yes , No |
| If yes, were you involved in developing the data management plans? | Yes , No |
| **2. Sample Handling and Storage Data** | |
| Did you have to make any changes to your normal laboratory data handling and storage practices for your involvement in VACCELERATE network?  **(You can compare this to your involvement with other multi-site clinical trials.)** | Yes , No  If yes, describe briefly |
| For samples received at your laboratory – how do you record the associated processing data, e.g. paper form, record on local database, both? | Describe briefly: |
| For samples received at your laboratory – describe briefly how you record laboratory processing data on the trial database (eCRF)? | Describe briefly: |
| For laboratory processing data recorded in the trial database (eCRF) – how do you reconcile queries with CTCC? Please outline steps involved? | Describe briefly: |
| Do you have any additional comments on how Vaccelerate sample handling and storage data could be managed? |  |
| **3. Data Transfer** | |
| Did you have to make any changes to your normal laboratory data transfer practices for your involvement in VACCELERATE network?  **(You can compare this to your involvement with other multi-site clinical trials.)** | Yes , No  If yes, describe briefly |
| Was any form of “data transfer agreement” suggested for use with the Vaccelerate trials, that lists for example, file format, naming conventions, encryption levels, method and type of transfer, recipient, test names, etc., | Yes , No |
| For laboratory providing analytics data – what format do you store data prior to transfer? | Describe briefly: |
| For laboratory providing analytics data – how do you transfer data to CTCC? What format and process are involved? | Describe briefly: |
| For laboratory providing analytics data – how do you reconcile queries with CTCC? Please outline steps involved? | Describe briefly: |
| For laboratory providing analytics data – how do you transfer data to Central Biobank? What format and process are involved? | Describe briefly: |
| For laboratory providing biobank facility – what format do you store data prior to transfer? | Describe briefly: |
| For laboratory providing biobank facility – how do you transfer data to CTCC? What format and process are involved? | Describe briefly: |
| For laboratory providing biobank facility – how does CTCC transfer data to you? What format and process are involved? | Describe briefly: |
| For laboratory providing biobank facility – how do you reconcile queries with CTCC? Please outline steps involved? | Describe briefly: |
| Do you have any additional comments on how VACCELERATE data transfers could be managed? |  |
| **4. General Laboratory Data Management** | |
| Would you consider that you had to make significant changes to your Laboratory data management practices because of involvement in VACCELERATE network? | Yes , No  If yes, describe briefly |
| What data management and systems recommendations would you make for future Vaccelerate trials? | Describe briefly: |
